# Comparative and Subtractive Genomics Analysis of Multidrug‐Resistant *Klebsiella pneumoniae* Strains for Novel Target Identification and Drug Repurposing Strategies

**DOI:** 10.1155/ijm/1460586

**Published:** 2026-09-06

**Authors:** Manasi Bora, Prokriti Paul, Sanchaita Rajkhowa, Harshita Sonar, Borakha Bura Gohain, Magdi E. A. Zaki, Dhamodharan Prabhu

**Affiliations:** ^1^ Centre for Biotechnology and Bioinformatics, Dibrugarh University, Dibrugarh, Assam, India, dibru.ac.in; ^2^ Department of Chemistry, Imam Mohammad Ibn Saud Islamic University (IMSIU), Riyadh, Saudi Arabia, imamu.edu.sa; ^3^ Centre for Drug Discovery, Department of Biotechnology, Karpagam Academy of Higher Education, Coimbatore, Tamil Nadu, India, kahedu.edu.in

**Keywords:** comparative genomics, drug repurposing, *Klebsiella pneumoniae*, MDR strains

## Abstract

The rapid rise of multidrug‐resistant (MDR) *Klebsiella pneumoniae* has created a major global health challenge due to the limited availability of conserved therapeutic targets effective across diverse resistant strains. In this study, an integrative computational target‐discovery and drug‐repurposing framework was applied to six clinically relevant *K. pneumoniae* strains. Comparative genomic analysis identified 3012 conserved genes, which were subsequently filtered to nine essential, non‐host homologous proteins. Among these, three conserved cytoplasmic proteins (*accD*, *cpxR*, and *mraZ*) were prioritized for functional analysis, with acetyl‐CoA carboxylase subunit beta (*accD*) emerging as the most promising therapeutic target based on sequence conservation, predicted essentiality, subcellular localization, and pathway association. Structural assessment supported the reliability of the predicted *accD* model, whereas consensus binding‐site analysis identified key residues suitable for ligand interaction. Virtual screening of FDA‐approved drugs followed by molecular docking identified several compounds with favorable binding profiles toward *accD*. Subsequent molecular dynamics simulations, including root mean square deviation (RMSD), root mean square fluctuation (RMSF), radius of gyration (Rg), hydrogen‐bond occupancy, principal component analysis (PCA), and PCA‐based free energy landscape (FEL) analyses, consistently identified tenapanor, micafungin, deferoxamine, and cobicistat as the most stable protein‐ligand complexes, with tenapanor exhibiting the most favorable overall structural and thermodynamic stability profile. These findings identify *accD* as a promising therapeutic target in MDR *K. pneumoniae* and suggest several FDA‐approved compounds as potential candidates for drug repurposing. Although experimental validation is needed to confirm their biological activity and therapeutic potential, this study demonstrates the potential of integrating comparative genomics with molecular dynamics analyses to support antimicrobial target identification and drug repurposing against MDR bacterial pathogens.

## 1. Introduction

The increasing spread of antibiotic‐resistant microorganisms has become a serious health concern for global public health system, particularly in hospitals. *Klebsiella pneumoniae* is a gram‐negative facultative bacterium belonging to the Enterobacteriaceae family; it is one of them and is widely regarded as a clinically significant multidrug‐resistant (MDR) organism. It commonly causes infections including bloodstream infections, pneumonia and urinary tract infections, especially in immunocompromised individuals and long‐term hospitalized patients [1]. Recently, *K. pneumoniae* has become a growing concern because of its resistance to antibiotics.

The thick polysaccharide capsule of *K. pneumoniae* offers an important role in helping the bacterium to escape host immune defenses. In addition, the organism may gain resistance genes via horizontal gene transfer due to its extremely flexible genome [2]. Genetic elements like transposons, plasmids, and integrative conjugative components facilitate the rapid exchange of resistance determinants among members of the Enterobacteriaceae, leading to the widespread emergence of the rising incidence of antibiotic‐resistant microorganisms has emerged as a serious challenge for public health systems across the world, particularly within hospital environments [3]. Classical MDR and hypervirulent *Klebsiella pneumoniae* (hvKP) are generally considered distinct clinical forms of *K. pneumoniae*. MDR strains are typically associated with healthcare‐associated infections and are characterized by the presence of multiple antimicrobial resistance determinants [4]. In contrast, hvKP strains are commonly associated with increased virulence and possess factors such as hyper capsule production, hypermucoviscosity, and efficient iron‐acquisition systems, which contribute to their ability to cause invasive infections including liver abscesses, meningitis, and endophthalmitis, even in otherwise healthy individuals [1, 5]. More recently, reports have described the emergence of strains that combine both multidrug resistance and hypervirulent characteristics, raising concerns regarding treatment options and clinical management [6]. The World Health Organization (WHO) has declared *K. pneumoniae* as a “critical priority pathogen” in the Indian Priority Pathogen List (IPPL) 2024 based on its clinical importance, mainly resistance to multiple antibiotics, frequent involvement in hospital‐settings infections, and the limited availability of effective treatment options [7]. Despite appropriate antimicrobial treatment, infections caused by *K. pneumoniae* are still associated with high rates of mortality, due to the continuous emergence of antimicrobial resistance and the limited availability of well‐characterized bacterial targets for therapeutic intervention. Traditional drug discovery methods can frequently be time‐consuming, costly, and have failure rates in the early stages of development.

Because of this, computational approaches are now broadly used for accelerating the investigation of ideal drug targets, particularly in MDR pathogens as they help in rapidly identifying new drug targets. Methods such as genome subtraction and genome comparison allow careful analysis of pathogenic genomes to identify essential and conserved proteins that are not present in the human host [8, 9]. Targeting these proteins helps reduce the risk of toxicity in host and unwanted side effects, while improving the specificity of antimicrobial agents [10]. In addition, comparing multiple strains ensures that the selected targets are conserved, increasing their usefulness for developing broad‐spectrum therapies.

However, the reliability of computational predictions may be influenced by factors such as genome annotation quality, database completeness, and the accuracy of structural models. Therefore, computationally identified targets should be considered preliminary candidates that require further biological and experimental validation to confirm their suitability for therapeutic development [11]. Furthermore, the high genetic diversity observed among MDR and hvKP strains can limit the applicability of strain‐specific therapeutic targets. Therefore, identifying conserved essential proteins that are maintained across genetically diverse clinical lineages is important for developing broadly effective therapeutic interventions.

Proteins present in the cytoplasm and involved in basic metabolic processes or gene regulation are often chosen as targets for new drugs because they are essential for the survival of bacteria [12]. Tools like RAST, Prokka, and CGView help to identify the functions of these bacterial proteins. Proteins that are similar to human or gut microbiome proteins are removed to narrow down the list of potential targets. Lastly, structure‐based computational methods, such as examining protein structures, predicting binding sites, molecular docking, and molecular dynamics (MD) simulations, can be employed to retrieve existing FDA‐approved drugs that may be repurposed. This approach offers a faster and more cost‐effective way to develop new antimicrobial treatments [13].

Although several studies have applied subtractive genomics approaches to identify potential therapeutic targets in *K. pneumoniae*, many have focused on individual strains, limited datasets, or target identification alone without extensive structural and drug‐repurposing analyses [14, 15]. In contrast, the present study integrates comparative genomics across six clinically significant MDR *K. pneumoniae* strains with subtractive proteomics, structural validation, binding‐site characterization, virtual screening of FDA‐approved compounds, and MD simulations. This comprehensive strategy enables the identification of highly conserved therapeutic targets and the prioritization of repurposable drug candidates with potential applicability across genetically diverse *K. pneumoniae* lineages.

In this context, the current study is aimed at identifying a conserved and essential therapeutic target shared across six clinically important MDR *K. pneumoniae* strains representing diverse epidemiological backgrounds. By focusing on conserved proteins maintained across genetically distinct strains, this study seeks to address the challenges posed by strain heterogeneity and improve the likelihood of identifying broadly applicable therapeutic targets. The study is also focused on repurposing existing approved drugs through structure‐based docking, interaction analysis, and MD simulations. By combining genome comparison, genome subtraction, pathway analysis, structure‐based virtual screening and MD simulations, this research is aimed at uncovering potential drug candidates that can be quickly advanced for preclinical testing against MDR *K. pneumoniae.*

## 2. Methods and Methodology

**Figure 1:**
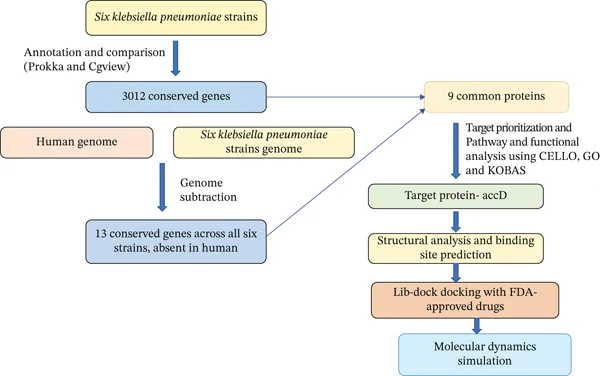
Workflow of the study.

Stepwise computational method was utilized to determine the potential drug target in MDR *K. pneumonia*. The overall strategy is displayed in Figure 1, which illustrates the workflow of genome comparison and subtraction used in this study. The method was categorized into three main steps: identifying suitable drug targets, using structure‐based virtual screening to test candidate compounds, and performing MD simulations to study drug repurposing and stability.

In the drug target identification phase, initially the genome of six clinically relevant MDR *K. pneumoniae* strains was taken from the National Center for Biotechnology Information (NCBI) and analyzed to find the gene common across all the strains. A subtractive genomics method was then implied to exclude human homologs, yielding nonhomologous genes consistently present in all six strains but absent in humans. From this, consensus essential proteins were investigated as potential therapeutic targets.

In the initial phase, FDA‐approved drugs from the DrugBank database were virtually selected against the most promising target, enabling the identification of favorable target‐drug interactions. This approach ultimately highlighted potential repurposable drugs effective across all six MDR *K. pneumoniae* strains.

### 2.1. Retrieval of Genomic Data for MDR *K. pneumoniae* Strains and the Human Host

To perform the comparative and subtractive genomic analysis, complete genome assemblies of six clinically significant MDR *K. pneumoniae* strains were obtained from the NCBI GenBank database (https://www.ncbi.nlm.nih.gov/). The selected strains included one reference genome, *K. pneumoniae* HS11286 (available under NCBI Accession Numbers NC_016845.1 and NC_016846.1), a well‐characterized strain from China widely used in genomic studies, and five MDR clinical isolates collected between 2020–2025 selected based on their clinical importance, high prevalence of carbapenem and colistin resistance, and representation of distinct high‐risk clones associated with nosocomial outbreaks. The strain VA585‐22 (CP135186.1), isolated in 2022 from a ventilated pneumonia patient in Chile, represents a recent and highly resistant South American isolate [16]. ST307 (CP103687.1), first reported in the Netherlands in 2008, is now a globally disseminated high‐risk clone associated with outbreaks across Europe and North America [17]. ST231 (NZ_CANDXP010000001.1), a carbapenem‐resistant lineage, has been predominantly reported in India and Southeast Asia, where it poses a significant clinical burden [18]. ST11 (CANDYF010000001.1), among the most globally dominant clones, is frequently encountered in Asia and Latin America and is recognized for its high virulence and epidemic potential [3]. ST16 (CANDXR010000001.1), an emerging clone implicated in nosocomial outbreaks worldwide, exhibits increasing multidrug resistance and virulence traits [19].

Sequence types (STs) ST11, ST16, ST231, and ST307 have been widely recognized as high‐risk clones due to their frequent association with hospital‐acquired infections, antimicrobial resistance, and successful international spread. Among these, ST11 is one of the most prevalent carbapenem‐resistant *K. pneumoniae* lineages, particularly in Asia, and has been increasingly associated with the convergence of antimicrobial resistance and hypervirulence traits [8, 20]. ST307 has emerged as a globally disseminated MDR clone frequently linked to extended‐spectrum *β*‐lactamase (ESBL) production and carbapenem resistance, contributing to numerous healthcare‐associated outbreaks worldwide [2, 9]. Similarly, ST231 has been identified as an important carbapenem‐resistant lineage carrying clinically relevant resistance determinants, including blaOXA‐232 and blaNDM variants, and has been increasingly reported across several geographic regions [3, 10]. Although less extensively studied than ST11 or ST307, ST16 has also been associated with multidrug resistance and nosocomial outbreaks, highlighting its clinical relevance as an emerging high‐risk lineage [7, 12].

In addition to these STs, strain HS11286 was included as a well‐characterized reference genome that has been widely used in genomic and comparative studies of *K. pneumoniae* due to its extensive annotation and documented MDR phenotype [13]. The recently isolated strain VA585‐22 was incorporated to provide representation of contemporary clinical isolates and to broaden the genomic diversity captured in the analysis. Collectively, the inclusion of these strains was intended to encompass genetically diverse yet clinically significant MDR *K. pneumoniae* lineages, thereby increasing the likelihood of identifying conserved therapeutic targets with potential applicability across multiple epidemiologically relevant strains.

To facilitate non‐homology based genome subtraction, the human reference proteome dataset (GCF_000001405.40, GRCh38.p14) was obtained from the NCBI RefSeq database. Furthermore, the Database of Essential Genes (DEG) (http://origin.tubic.org/deg/public/index.php) [21] was accessed to retrieve curated lists of experimentally validated essential proteins across a broad range of bacterial species. The DEG was used as a reference as it contains experimentally validated essential genes from a wide range of bacterial species, allowing the identification of proteins that are likely to be important for bacterial survival. These essential genes serve as a foundational filter to prioritize proteins that are indispensable for bacterial survival and thus represent viable antimicrobial targets. All genomic and proteomic datasets were downloaded in standard FASTA format and processed locally for downstream computational analyses.

### 2.2. Functional Annotation Using RAST

Rapid Annotations Employing Subsystems Technology (RAST) was used for functional annotation of six *K. pneumoniae* strains: HS11286, VA585‐22, ST231, ST307, ST11, and ST16. The genome sequences were uploaded to the RAST server in FASTA format for annotation (http://RAST.nmpdr.org) [22]. Gene prediction was performed using the GLIMMER3 algorithm, and the annotated output included coding sequences (CDSs), transfer RNAs (tRNAs), ribosomal RNAs (rRNAs), and their associated functional subsystems. These subsystems comprised genes related to virulence, antibiotic resistance, and cellular metabolism. These annotations were used for further analysis and helped identify genes related to disease and drug resistance.

### 2.3. Visualization of Genome and Comparative Genomics Using CGView and Prokka

For visualization of genomic organization, genome annotation and comparison was performed using Prokka, and annotated files were subsequently used to generate circular genome maps with CGView [23, 24]. Prokka employed annotation tools such as Prodigal, Aragorn, and RNAmmer, producing output files in GFF, GBK, and FASTA formats. CGView was then used to visualize GC content, gene distribution, and genomic islands, allowing clear comparison of conserved and strain‐specific genomic features across the six *K. pneumoniae* genomes.

### 2.4. Subtractive Genomics Approach

To identify conserved, essential, and non‐host homologous targets for *K. pneumoniae*, a genome subtraction method was employed. Complete proteomes of six *K. pneumoniae* strains (HS11286:ASM24018v2 https://www.ncbi.nlm.nih.gov/datasets/genome/GCF_000240185.1/, VA585‐22:ASM3225393v1 https://www.ncbi.nlm.nih.gov/datasets/genome/GCF_032253935.1/, ST11:17‐8104547_assembly https://www.ncbi.nlm.nih.gov/datasets/genome/GCF_947391185.1/, ST231:17‐8104516_assembly https://www.ncbi.nlm.nih.gov/datasets/genome/GCF_947390235.1/, ST16:17‐8104601_assembly https://www.ncbi.nlm.nih.gov/datasets/genome/GCF_947390345.1/, ST307: ASM2491845v1 (28) *Homo sapiens*; GRCh38.p14 https://www.ncbi.nlm.nih.gov/datasets/genome/GCF_000001405.40/) were retrieved from NCBI database. Initially, CD‐HIT (v4.8.1) [25] was used to cluster and eliminate paralogous sequences at a 90% identity threshold, ensuring non‐redundancy in each proteome. The resulting non‐redundant protein sequences were passed through BLASTp analysis against the human proteome, with an 1e‐5 E‐value threshold and sequence identity cutoff < 25%, to filter out proteins homologous to the host. Proteins showing significant similarity to the human proteome were excluded to reduce the possibility of off‐target effects and improve the specificity of the identified therapeutic targets. The BLASTp threshold used in this study was selected in accordance with commonly applied criteria in subtractive genomics studies. The final pool of unique and non‐host homologous proteins are then analyzed by TBLASTN against the DEG, applying a stringent bit score > 75 and E‐value ≤ 1e‐5 to identify proteins essential for bacterial survival and viability.

To further evaluate their therapeutic importance, the protein sequences were mapped to UniProt identifiers using the UniProt ID Mapping tool (http://www.uniprot.org/) [26]. These UniProt entries were then checked against the DrugBank database to see whether any of the identified proteins were already associated with existing drugs.

### 2.5. Target Validation Using DrugBank

Each essential protein was searched against the DrugBank database (https://go.drugbank.com/) to check its potential to be targeted by drugs [27]. Proteins that showed similarity to known drug targets were identified as previously targeted. This step helped distinguish new targets from known ones. This step helped differentiate novel from previously targeted proteins and served as the basis for repurposing FDA‐approved drugs.

### 2.6. Subcellular Localization Prediction

CELLO v2.5 (http://cello.life.nctu.edu.tw/cello2go/) was used to predict the subcellular localization of the shortlisted proteins [28]. Cytoplasmic proteins were prioritized for further screening, as they are more amenable to small‐molecule targeting, whereas membrane‐bound or extracellular proteins were considered for vaccine design [29].

### 2.7. Pathway Annotation and Enrichment via GO‐PANTHER and KOBAS

Functional roles and pathway involvements of candidate proteins were assessed using Gene Ontology (GO) annotations from the PANTHER database (http://www.geneontology.org/) [30]. Biological processes (BP), molecular functions (MFs), and cellular components (CCs) were mapped. For enrichment analysis, KOBAS 3.0 (http://kobas.cbi.pku.edu.cn.) was employed to identify statistically significant KEGG pathways using hypergeometric testing and Benjamini–Hochberg false discovery rate (FDR) correction [31, 32]. Statistical significance was assessed using the Benjamini–Hochberg FDR correction implemented in KOBAS 3.0 to account for multiple hypothesis testing. Pathways with corrected *p* values < 0.05 were considered statistically significant, whereas pathways with corrected *p* values between 0.05 and 0.10 were interpreted as suggestive evidence and evaluated together with complementary biological analyses.

### 2.8. Structural Validation and Superimposition

The AlphaFold‐predicted 3D structure of the final target protein acetyl‐coenzyme A carboxylase carboxyl transferase (accD) was obtained from the AlphaFold Protein Structure Database via UniProt (Accession ID: [A0A0H3GT58]). As there was no experimentally resolved crystal structure for accD from *K. pneumoniae* available in the Protein Data Bank (PDB), a homology‐based validation approach was undertaken to assess the reliability of the AlphaFold‐predicted model. A BLASTp search was conducted against the PDB database using BLAST+ [33] with default parameters to identify closely related homologs with experimentally determined structures (ftp://ftp.ncbi.nlm.nih.gov/blast/executables/blast+/LATEST). Among the retrieved hits, the top structural homolog was selected based on sequence similarity, alignment score, and structural relevance. To validate the predicted tertiary structure, a structural superimposition was performed between the AlphaFold model and the selected PDB homolog using Discovery Studio Visualizer (Dassault Systèmes BIOVIA). The root mean square deviation (RMSD) was calculated to measure the structural similarity between the two models. When the RMSD value is low, it indicates that the AlphaFold‐predicted structure closely matched to the experimentally determined structure, indicating that the predicted model was reliable. This validation step confirmed that the structure was accurate and suitable for docking and ligand interaction studies.

### 2.9. Binding Site Prediction Using CASTp and FTMap

CASTp and FTMap are two complementary computational tools used to identify possible ligand‐binding regions on the validated 3D structure of the accD protein. CASTp (Computed Atlas of Surface Topography of Proteins; https://cfold.bme.uic.edu/castpfold/) was used to examine the surface topology of the accD structure and to identify solvent‐accessible pockets and internal cavities. This analysis provided information on pocket size and surface area, helping to locate regions that can bind small molecules [34].

At the same time FTMap (http://ftmap.bu.edu/param/) was used as a fragment‐based binding site prediction method to locate energetically favorable binding regions. FTMap uses a probe‐based docking approach in which multiple small organic fragments are docked onto the protein surface to detect consensus regions with low binding free energy. These regions represent potential ligand‐binding “hotspots” that are critical for high‐affinity interactions [35].

After both analyses were completed, the binding sites predicted by CASTp and FTMap were compared. The overlap between the two methods increased confidence in the selected binding pocket and ensured that it was suitable for further structure‐based drug repurposing studies.

### 2.10. Molecular Docking Using Discovery Studio

Lib‐Dock module in Discovery Studio was used to carry out structure‐based virtual screening [36]. Lib‐Dock can screen vast libraries of compounds and predict how they fit into the protein binding location. The method uses precalculated polar and nonpolar interaction grids to place ligands in the binding site and check how well they fit. The validated 3D model predicted by AlphaFold was used as the receptor for docking studies. The binding site was defined based on the predictions obtained from CASTp and FTMap.

A library of FDA‐approved small‐molecule drugs was retrieved, prepared, and energy‐minimized. Prior to molecular docking, all ligand structures were prepared using the Prepare Ligands protocol available in BIOVIA Discovery Studio. Hydrogen atoms were added, bond orders were assigned, and the ligand geometries were energy‐minimized using the CHARMm force field to obtain optimized conformations suitable for docking. Each ligand was docked into the active site, and Lib‐Dock generated multiple poses, scoring them based on their steric and electrostatic fit within the pocket.

Top‐ranked compounds were selected based on Lib‐Dock scores, binding energies, and the nature of key molecular interactions, including hydrogen bonds and hydrophobic contacts. These ligands were prioritized for further evaluation as potential repurposed drug candidates targeting MDR *K. pneumoniae*.

### 2.11. MD Simulations

MD simulations were performed on the seven most promising compounds predicted on the basis of strong binding energy during docking analysis. Here, the unbound native protein was served as a reference control, and the best‐docked poses were considered as the starting structures for the respective MD simulations. Overall total, 800 ns of MD simulations were carried out, with each protein‐ligand complex given to a 100 ns run using the GROMACS 2024 package [37, 38]. Ligand topologies were constructed by the automated topology builder (ATB), which incorporates the GROMOS 54a7 force field [39]. Each complex was placed in a cubic simulation box solvated with SPC/E water, maintaining a minimum distance of 1.0 nm between the protein surface and the box edges. Energy minimization was first performed using the steepest descent algorithm. The systems were then equilibrated in two stages: 3 ns under NVT conditions for temperature stabilization, followed by 5 ns under NPT conditions to maintain a temperature of 300 K and a pressure of 1 bar [32, 40]. Following equilibration, 100 ns production MD simulations were carried out for each system. Simulation stability was assessed by monitoring the convergence of the RMSD trajectories together with root mean square fluctuation (RMSF), radius of gyration (Rg), and hydrogen‐bond profiles throughout the simulation. Stable RMSD profiles with no major structural deviations were considered indicative of equilibrated protein‐ligand complexes [41]. The Xmgrace program was used for data visualization and graphical representation of the simulation results.

### 2.12. Free Energy Landscape (FEL) Analysis

FEL analysis was performed as the final post‐MD simulation analysis to investigate the thermodynamic stability, conformational dynamics, and energetically favorable conformational states of the native accD protein and its ligand‐bound complexes. This analysis was carried out following the evaluation of structural stability using RMSD, RMSF, Rg, hydrogen‐bond analysis, hydrogen‐bond occupancy, and principal component analysis (PCA). FEL analysis provides a thermodynamic representation of the conformational space explored by the protein–ligand complexes during the 100 ns MD simulations [42].

Prior to FEL generation, the simulation trajectories were processed using the gmx trjconv module of GROMACS 2024 to remove periodic boundary condition (PBC) artifacts. The protein was centered within the simulation box using the ‐center and ‐pbc mol options, followed by least‐squares fitting of the protein backbone to the initial reference structure to eliminate overall translational and rotational motions [43]. This preprocessing ensured that only intrinsic conformational changes of the protein‐ligand complexes were retained for subsequent analyses.

PCA‐based FELs were constructed using the first two principal components (PC1 and PC2) as reaction coordinates, as these components captured the dominant collective motions of the simulated systems. The covariance matrix of the protein backbone atomic coordinates was generated using the gmx covar module, and the corresponding eigenvectors and principal components were calculated using gmx anaeig. Subsequently, the gmx sham module was employed to calculate the relative Gibbs free energy and generate both two‐dimensional contour maps and three‐dimensional (3D) free energy surface plots based on PC1 and PC2. In the resulting FELs, deep and localized energy minima represent thermodynamically stable conformational states, whereas broader or multiple energy basins indicate increased conformational flexibility and transitions between metastable states [44].

## 3. Results

This study employed an integrated comparative and subtractive genomics approach to systematically identify conserved therapeutic targets in MDR *K. pneumoniae*. The workflow prioritized proteins that are essential for bacterial survival, conserved across clinically relevant strains, and non‐homologous to human proteins, thereby minimizing the potential for host toxicity and off‐target effects. The prioritized targets were subsequently subjected to structural characterization, virtual screening, and MD analyses to identify potential repurposable drug candidates [8, 10].

### 3.1. Functional Annotation and Comparative Genomic Profiling Using RAST and CGview

#### 3.1.1. RAST Analysis

**Figure 2:**
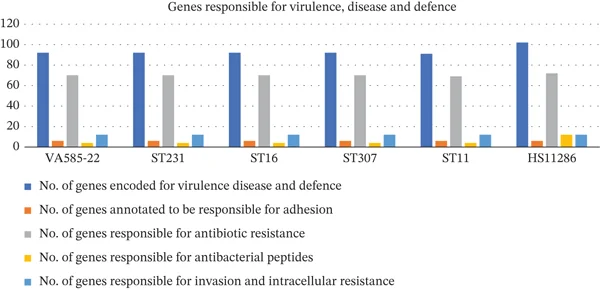
Functional categorization of virulence‐associated genes in six *K. pneumoniae* strains using RAST annotation.

Subsystem‐based categorization revealed a broadly conserved distribution of genes associated with amino acid biosynthesis, carbohydrate metabolism, membrane transport, and virulence‐related processes across all strains (Figure S1; SI Table S1). Most importantly, enrichment of genes linked to iron uptake, quorum sensing as well as antibiotic resistance was frequently observed (Figure 2). This indicates the isolates′ inherent hypervirulence and multidrug resistance. These findings are consistent with earlier analysis that demonstrate the key role in which iron absorption methods and metabolic flexibility play in *K. pneumoniae* pathogenicity and survival under antimicrobial stress. Crucial annotated categories from all six genomes are summarized in the bar graph shown in Figure 2.

The distribution of genes classified under the “Virulence, Disease, and Defense” subsystem for each of the six *K. pneumoniae* strains evaluated is displayed in Figure 2. A comparative study of the “Virulence, Disease, and Defence” subsystem found substantial conservation in genes associated with intracellular survival, adhesion, and antimicrobial peptide resistance, indicating the existence of a core set of virulence determinants. This result bolsters the viability of discovering broad‐spectrum targets for therapeutic intervention that are preserved among resistant strains [45].

#### 3.1.2. CGView–Prokka Analysis

The genomic organization of the selected *K. pneumoniae* strains was visualized using CGView with Prokka‐based annotations. The circular genome maps showed a similar overall genome organization across all strains, including comparable distributions of tRNA clusters and rRNA operons. However, there were also apparent variable areas, particularly among bacteria that were MDR. These areas most likely suggest genomic islands or prophage insertions and point to the possibility of horizontal gene transmission, a crucial process. This method underlies *K. pneumoniae*′s development of virulence characteristics and antibiotic resistance [46].

**Figure 3:**
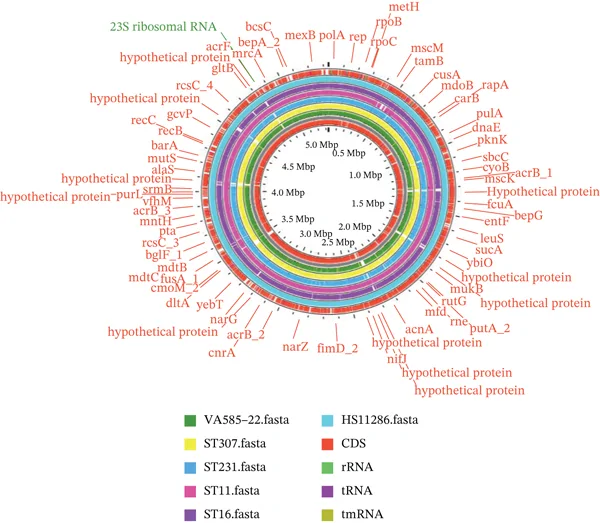
Circular comparative genome map of *K. pneumoniae* strains generated using CGView.

To examine the genomic organization and enable comparison among the selected strains, the annotated genome files were visualized using CGView, as shown in Figure 3.

The annotated genome of the reference strain HS11286 was placed prominently in the circular map, with concentric rings reflecting the remaining five strains′ BLAST‐based alignments (ST11, ST16, ST231, ST307, and VA585‐22). The inner layers displayed GC content and GC skew profiles, as the outside rings showed Prokka‐identified CDSs, tRNAs, and rRNAs (Figure 3). The interaction among the evolutionary conservation and adaptive genomic plasticity in MDR clinical isolates was demonstrated by this image. Additionally, it made it possible to identify not only the conserved genomic backbones but also strain‐specific variable regions.

**Figure 4:**
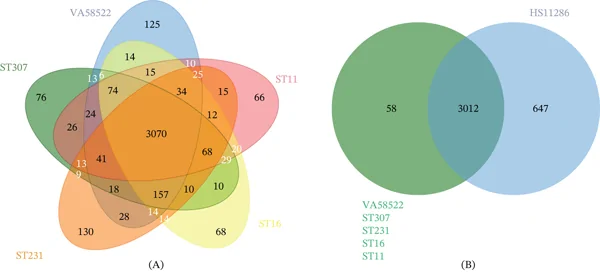
A Venn diagram based on Prokka annotations showing common genes between six strains of *K. pneumoniae*. (A) Five strains (VA585‐22, ST231, ST307, ST11, and ST16) are compared. (B) Extended comparison with reference strain HS11286.

Annotated gene data from Prokka has been examined to figure out the shared genomic content of all six strains of *K. pneumoniae*, and the common genes are displayed using a Venn diagram shown in Figure 4.

Figure 4A represents the intersection of annotated gene sets from the five selected *K. pneumoniae* strains (ST11, ST16, ST231, ST307, and VA585‐22), whereas Figure 4B represents the intersection of the common gene sets from these five selected strains with the most annotated strain, HS11286, which was considered as the reference genome. A total of 3012 genes were found to be conserved across all strains, indicating a core genome that may encode essential functions and serve as a foundation for downstream subtractive genomics and drug target identification. Strain‐specific and variably conserved genes, represented in the non‐overlapping regions of the diagram, likely encode functions associated with niche adaptation, virulence modulation, and antimicrobial resistance. Similar approaches integrating Prokka‐based annotations with comparative visualization have been successfully applied in related studies, such as in the comparative genome analysis of *Lactobacillus paracasei* [47].

### 3.2. Subtractive Genomics Approach

The core proteome of each strain was individually filtered through the human proteome using BLASTp to eliminate homologous sequences, ensuring selection of non‐host proteins as drug targets. Approximately 3200–3400 proteins were predicted per strain, out of which 1343 proteins were common and non‐homologous to the human host. The subtractive genomics pipeline yielded 13 proteins that were conserved across all six *K. pneumoniae* strains and exhibited no sequence similarity with the human proteome. These proteins were then subjected to essentiality assessment using the DEG database, suggesting their involvement in vital bacterial processes such as peptidoglycan biosynthesis, lipid metabolism, and regulatory signaling.

**Figure 5:**
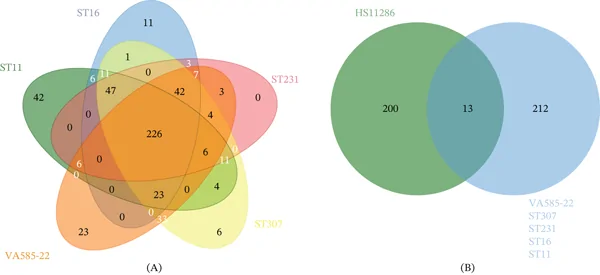
Venn diagram showing the distribution of essential genes among six *K. pneumoniae* strains following subtractive genomics analysis. (A) Comparison of essential genes among VA585‐22, ST231, ST307, ST11, and ST16 strains. (B) Comparison including the reference strain HS11286.

To visualize the overlap of essential non‐host homologous genes identified through genome subtraction, a Venn diagram (Figure 5) was constructed.

Figure 5A displays the overlap of essential, non‐host homologous genes identified across the five *K. pneumoniae* strains (ST11, ST16, ST231, ST307, and VA585‐22), whereas Figure 5B shows the extended comparison of the five strains with the reference genome, HS11286. A core set of essential genes common to all strains was identified, indicating conserved proteins crucial for bacterial survival. These shared targets represent promising candidates for broad‐spectrum antimicrobial drug development, whereas uniquely distributed genes suggest strain‐specific adaptations [40].

**Figure 6:**
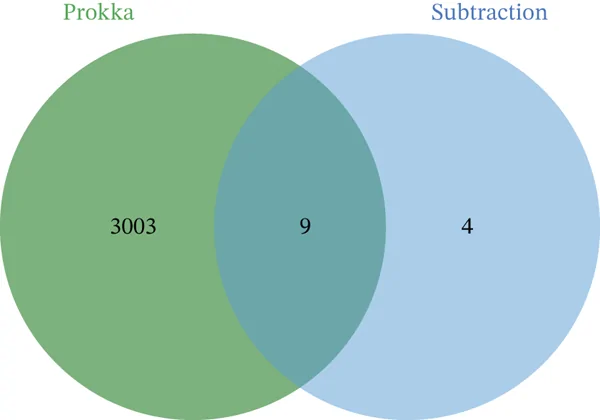
Venn diagram comparing the conserved proteins identified by Prokka with the essential non‐host homologous proteins obtained through subtractive genomics.

To ensure consistency between genome‐wide annotation and the subtractive genomics pipeline, the core proteome identified through Prokka (3012 conserved proteins across all six *K. pneumoniae* strains) was cross‐compared with the essential, non‐host homologous proteins obtained following subtractive filtering (Figure 6).

A total of nine overlapping proteins: bamD, accD, fadR, lolB, murJ, potF, dapD, mraZ, and cpxR were consistently detected across both approaches, representing high‐confidence candidates for drug target prioritization (Figure 6). These proteins represent not only core elements conserved across the six *K. pneumoniae* strains but also fulfill critical criteria of essentiality and non‐homology to the human proteome. The four genes absent from the Prokka annotation were excluded to maintain consistency across standardized genome annotation pipelines, thereby minimizing redundancy and eliminating low‐confidence predictions. This ensured that only reliably annotated and biologically relevant proteins were advanced for downstream analysis.

### 3.3. Target Evaluation and Sequence Conservation Analysis

The nine conserved essential non‐host homologous proteins were evaluated for therapeutic relevance using the DrugBank database, which catalogs FDA‐approved drugs and experimentally supported drug‐target interactions. Of these, four proteins were identified as previously characterized therapeutic targets with existing associations to approved or experimental therapeutics.

To further establish their suitability as broad‐spectrum targets, sequence conservation across all six *K. pneumoniae* strains was assessed using EMBOSS Needle, which applies the Needleman‐Wunsch global alignment algorithm. The analysis revealed consistently high sequence identity and similarity, supporting their evolutionary conservation. These findings suggest that the four proteins are conserved and represent promising candidates for therapeutic targets, with detailed conservation data summarized in Table 1. Similar approaches integrating subtractive proteomics and target screening have been successfully applied in other bacterial pathogens, reinforcing the reliability of this pipeline for novel antimicrobial target identification [48].

**Table 1:** Summary of nine essential non‐host homologous proteins with target evaluation, localization, and conservation data.

Gene	Protein name	Previously reported target	Localization	Target organism	Associated drug	Identity (%)	UniProt Id
accD	Acetyl‐coenzyme A carboxylase carboxyl transferase subunit beta	No	Cytoplasm	N/A	N/A	N/A	N/A
murJ	Probable lipid II flippase MurJ	No	Inner membrane	N/A	N/A	N/A	N/A
dapD	2,3,4,5‐tetra hydro pyridine‐2,6‐dicarboxylate N‐succinyl Transferase	Yes	Cytoplasm	Unknown prokaryotic organism	Coenzyme A, L‐2‐amino pimelic acid, pimelic acid, succinamide‐CoA, succinyl‐coenzyme A	N/A	P56 220
lolB	Outer‐membrane lipoprotein LolB	Yes	Cell outer membrane	*Shigella flexneri , Escherichia coli* (strain K12)	Triglyme	(S) 79.7	P61322,
						(E) 79.7	P61320
cpxR	Transcriptional regulatory protein cpxR	No	Cytoplasm	N/A	N/A	N/A	N/A
fadR	Fatty acid metabolism regulator protein	Yes	Cytoplasm	*Shigella flexneri, Bacillus subtilis* (strain 168), *Escherichia coli* (strain K12)	(S) Coenzyme A, (B) dodecyl‐Coa, (E) myristic acid	(S) 90.4	P0A8V,
						(B) 11.6	P94548,
						(S) 90.4	P0A8V9
							P0A8V6
potF	Putrescine‐binding periplasmic protein	Yes	Periplasm	*Escherichia coli* (strain K12)	Putrescine	32.6	P31
							133
bamD	Outer membrane protein assembly factor BamD	No	Periplasm	N/A	N/A	N/A	N/A
mraZ	Transcriptional regulator mraZ	No	Cytoplasm	N/A	N/A	N/A	N/A

### 3.4. Identification of Subcellular Localization

Following the identification of nine essential non‐host homologous proteins conserved across the six *K. pneumoniae* strains, subcellular localization was predicted using CELLO v2.5, a machine‐learning‐based tool that classifies bacterial proteins into defined cellular compartments [49].

The enriched GO terms were ranked based on their statistical significance to identify the most biologically relevant functions (Tables S2A–C). Several significantly enriched terms were associated with BP involved in bacterial growth, metabolism, transcriptional regulation, and stress response. The MF category highlighted enzymatic activities, including ligase, transferase, and kinase functions, suggesting their roles in essential metabolic processes and bacterial survival under adverse conditions. The CC analysis further indicated that these proteins are predominantly localized in the cytoplasm, consistent with the subcellular localization predictions obtained using CELLO. Their cytoplasmic localization, together with their involvement in essential cellular functions, supports their potential as promising therapeutic targets [28].

### 3.5. Pathway Analysis Using GO Panther

GO enrichment analysis was conducted on the three prioritized target proteins using the PANTHER classification system [50]. The analysis revealed 48 significantly enriched BP, along with 16 MF and 5 CC, providing comprehensive functional annotations. Enriched terms were ranked by statistical significance to prioritize the most biologically relevant insights (Table S2A–C).

The predominant BP were associated with essential cellular functions, including fatty acid biosynthesis, transcriptional regulation, and stress response, which are key pathways involved in bacterial growth, adaptation, and virulence. The MF category highlighted enzymatic activities such as ligases, transferases, and kinases, indicating their roles in metabolic regulation and bacterial survival under adverse conditions [51]. CC analysis localized these proteins mainly to the cytoplasm, consistent with subcellular localization predictions from CELLO [28], thereby supporting their accessibility as potential therapeutic targets.

**Figure 7:**
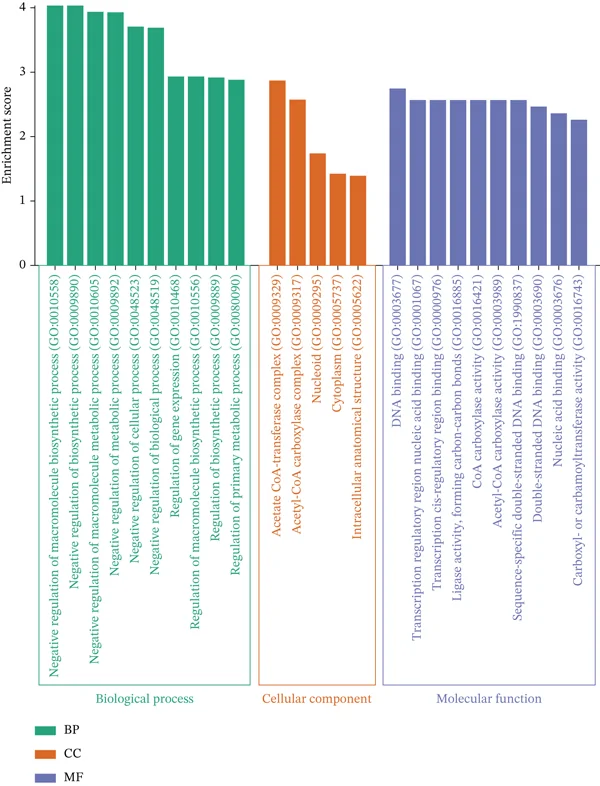
Gene Ontology (GO) enrichment analysis showing the top enriched terms across the biological process (BP), molecular function (MF), and cellular component (CC) categories.

The results of GO enrichment analysis, performed using the PANTHER classification system were visualized in the form of a bar graph, as shown in Figure 7.

The top enriched terms in all of the three GO categories such as BP, MF, and CC are displayed in Figure 7, which provides an in‐depth overview of the GO enrichment analysis results. The BP terms experienced the highest enrichment scores within these categories. The most essential enriched phrases were GO:0009890 (negative regulation of biosynthetic process) and GO:0010558 (negative regulation of macromolecule biosynthetic process). These findings suggest a possible involvement of the prioritized cytoplasmic proteins in metabolic regulation and bacterial survival processes. This also demonstrates their significant engagement in regulatory pathways connected to biosynthesis suppression.

The highest abundant phrase in the CC category was GO:0009329 (acetate‐CoA transferase complex), suggest that the proteins may be associated with particular enzyme complexes involved in intermediate metabolism. GO:0003677 (DNA binding) is the highest enrichment score for the MF category, which indicates an important role in nucleic acid interactions and regulatory mechanisms [52]. All of these results confirm the projected targets potential as antimicrobial targets by demonstrating their functional value in transcriptional regulation, metabolic control and essential enzymatic functions.

### 3.6. Pathway Enrichment Analysis Using KOBAS

KEGG pathway enrichment analysis was performed using the KOBAS 3.0 platform to investigate the biological functions and physiological significance of the shortlisted candidate proteins [31]. Among the three shortlisted proteins, accD and cpxR were associated with multiple KEGG pathways, whereas mraZ showed no annotated KEGG pathway enrichment. Table 2 summarizes the KEGG pathway enrichment results for the shortlisted proteins, including pathway names, KEGG IDs, corrected *p* values, and the corresponding mapped proteins.

**Table 2:** KEGG pathway enrichment analysis of target proteins using KOBAS.

Term	Database	ID	Input no.	Back‐ground no.	*p*	Corrected *p* value	Input
Fatty acid biosynthesis	KEGG PATHWAY	eco00061	1	13	0.009166246	0.071610941	accD
Fatty acid metabolism	KEGG PATHWAY	eco01212	1	21	0.014378864	0.071610941	accD
Cationic antimicrobial peptide (CAMP) resistance	KEGG PATHWAY	eco01503	1	36	0.024103185	0.071610941	cpxR
Propanoate metabolism	KEGG PATHWAY	eco00640	1	39	0.026040342	0.071610941	accD
Pyruvate metabolism	KEGG PATHWAY	eco00620	1	53	0.035046517	0.077102338	accD
Carbon metabolism	KEGG PATHWAY	eco01200	1	109	0.070516027	0.129279383	accD
Two‐component system	KEGG PATHWAY	eco02020	1	166	0.105715263	0.166123985	cpxR
Biosynthesis of antibiotics	KEGG PATHWAY	eco01130	1	210	0.132270441	0.181871856	accD
Microbial metabolism in diverse environments	KEGG PATHWAY	eco01120	1	266	0.165300719	0.202034212	accD
Biosynthesis of secondary metabolites	KEGG PATHWAY	eco01110	1	304	0.187229918	0.205952909	accD
Metabolic pathways	KEGG PATHWAY	eco01100	1	878	0.473318979	0.473318979	accD

As shown in Table 2, accD was associated with multiple metabolic pathways, including the biosynthesis of secondary metabolites and antibiotics, suggesting its broader functional involvement in bacterial metabolism. Similarly, cpxR was associated with the two‐component system and cationic antimicrobial peptide (CAMP) resistance pathways, consistent with its role in environmental sensing and antimicrobial resistance [53]. Although none of the pathways remained statistically significant after FDR correction (corrected *p* < 0.05), accD consistently showed the lowest corrected *p* values (~0.07) and the highest number of pathway associations among the shortlisted proteins. These results reinforce the established importance of fatty acid biosynthesis in bacterial viability and antibiotic susceptibility, as previously noted in *Mycobacterium tuberculosis* and *Escherichia coli* [54, 55].

**Figure 8:**
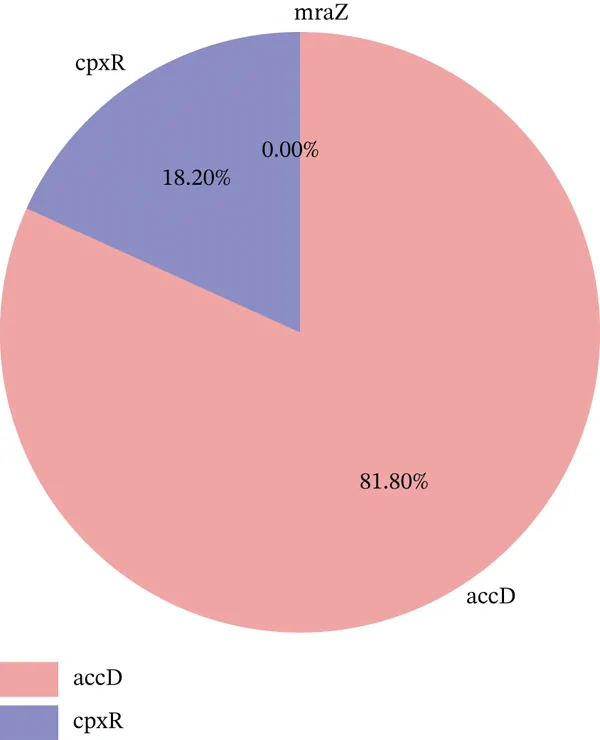
Distribution of genes across enriched KEGG pathways.

To visualize the relative functional contribution of each shortlisted protein in enriched KEGG pathways, a pie chart (Figure 8) was constructed based on the pathway enrichment results from KOBAS.

Figure 8 illustrates the percentage contribution of the three analyzed proteins accD, cpxR, and mraZ to the overall pathway enrichment results. The analysis revealed that accD accounted for the majority of the enriched pathways (81.80%), followed by cpxR (18.20%), whereas mraZ showed no pathway enrichment (0%). Taken together, the results of conservation analysis, subcellular localization, functional annotation, and pathway enrichment consistently prioritized accD over the other shortlisted proteins.

### 3.7. Sequence Alignment Using BLAST

To enable reliable molecular docking studies of the finalized target protein accD, a high‐resolution 3D structure was required. Since no experimentally determined crystallographic structure of *K. pneumoniae* accD was available in PDB, the AlphaFold‐predicted model obtained from UniProt was considered as an alternative structural template [56]. To validate the accuracy of this predicted model, a BLASTp search was conducted against the PDB using the accD amino acid sequence.

The analysis revealed a top‐scoring homolog, Chain B of acetyl‐coenzyme A carboxylase carboxyltransferase from *E. coli* (PDB ID: 2F9Y_B), which exhibited 97.06% sequence identity and 100% query coverage with the *K. pneumoniae* protein. This high degree of similarity supports the structural reliability of the AlphaFold model. Consequently, 2F9Y_B was selected as the reference template for subsequent structural superimposition. The use of PDB homologs to validate AlphaFold‐predicted protein structures has been widely adopted in recent computational drug discovery workflows [57], and the exceptionally high sequence identity further supports the suitability of the accD model for downstream structural and docking analyses.

The top‐scoring results of BLASTp analysis of the accD protein sequence from *K. pneumoniae* against the PDB database are summarized in Table 3.

**Table 3:** Top 5 results of the BLASTp analysis.

Description	Scientific name	Max score	Total score	Query cover	Per. identity	Accession
Chain B acetyl‐coenzyme A carboxylase carboxyl transferase subunit beta	*Escherichia coli*	603	603	100%	97.06%	2F9Y_B
Chain D acetyl‐coenzyme A carboxylase carboxyl transferase subunit beta	*Escherichia coli*	574	574	92%	98.94%	8UZ2_D
Chain B acetyl‐coenzyme A carboxylase carboxyl transferase subunit beta	*Staphylococcus aureus*	253	253	86%	46.59%	2F9I_B
Chain A methylmalonyl‐CoA carboxyltransferase 125 subunit	*Propioni bacterum freudenreichill*	93.2	93.2	74%	33.78%	1ON3_A
Chain A propionyl‐CoA carboxylase complex B subunit	*Streptomyces coelicolor*	78.6	78.6	63%	29.65%	1XNV_A

Table 3 summarizes the BLASTp results obtained for the accD protein sequence used to identify structurally characterized homologs in the PDB. The top hit, 2F9Y_B from *E. coli*, demonstrated the highest sequence identity (97.06%) and full query coverage, making it a suitable reference structure for superimposition and validation of the AlphaFold‐predicted accD model. This validation step ensured structural reliability for subsequent molecular docking studies.

### 3.8. Superimposition Using Discovery Studio

BIOVIA Discovery Studio Visualizer was employed to perform structural superimposition to check the correctness of the predicted 3D structure of the accD protein from *K. pneumoniae*. The reference crystal structure was determined from the top BLASTp hit from PDB, accession ID 2F9Y_B from *E. coli*. It revealed 97.06% sequence identity and full query coverage. The AlphaFold‐predicted structure and the PDB template RMSD of 0.532 Å after superimposition following the “Structure Superimposition” protocol in Discovery Studio. The low RMSD value supports the structural similarity between the AlphaFold model and the reference structure, suggesting a high range of structural alignment.

**Figure 9:**
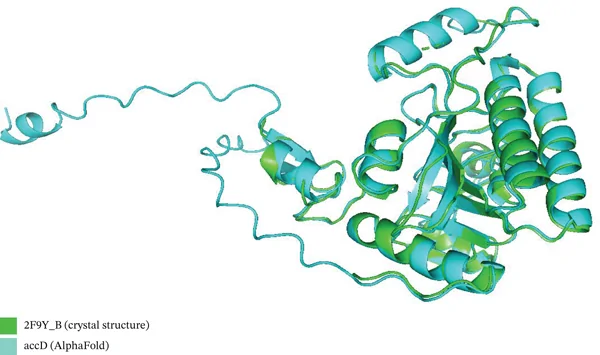
Superimposition of AlphaFold‐predicted structure of accD (cyan) with the PDB crystal structure 2F9Y_B from *Escherichia coli* (green).

Visual representation of the aligned structures is shown in Figure 9, further supporting the suitability of the predicted accD structure for subsequent molecular docking and interaction analyses.

Figure 9 shows a precise structural alignment between the AlphaFold‐predicted accD protein model and the experimentally determined crystal structure of its homolog. The high degree of overlap between the two structures, visually confirmed by the near‐perfect fitting of the green and cyan chains, supports the validity of the AlphaFold model. This close structural similarity, further quantified by an RMSD value of 0.532 Å, establishes confidence in using the predicted structure for downstream computational analyses such as active site identification and molecular docking.

### 3.9. Binding Site Prediction Using CASTpFold and FT‐Mapping

To identify the most probable binding pockets on the validated AlphaFold structure of the accD protein, two complementary tools CASTpFold and FTMap were employed. The integration of topology‐ and energy‐based predictions improves the reliability of pocket detection, as previously demonstrated in target evaluation studies of novel bacterial targets [58]. Importantly, this dual‐method validation increases the likelihood of successful docking and inhibitor design in subsequent stages. CASTpFold analyzed the 3D surface topology and identified key cavities based on volume and surface area, whereas FTMap used probe‐based mapping to detect energetically favorable ligand‐binding hotspots. A total of 99 interacting residues were predicted by FTMap and 19 residues by CASTpFold. Nineteen amino acids were found to be common between both tools.

**Figure 10:**
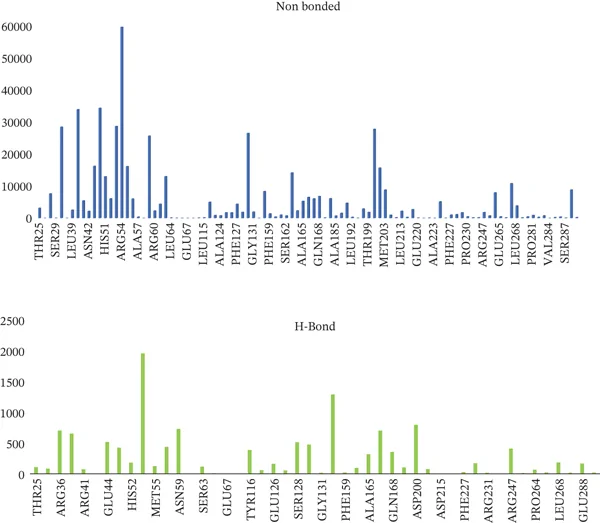
H‐bonded and nonbonded interaction of accD protein (alpha‐fold structure).

The interaction profile of the accD protein, as predicted by FTMap, is represented in Figure 10, highlighting the frequency of hydrogen‐bonded (H‐bonded) and nonbonded residues involved in potential ligand binding.

**Figure 11:**
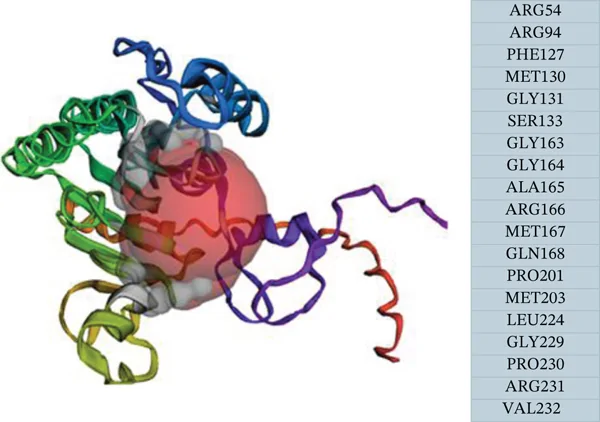
Predicted binding pocket of the *K. pneumoniae* accD protein identified by CASTpFold, showing the cavity topology and the 19 contributing residues.

Figure 10 highlights both H‐bonded and other nonbonded interactions with probe molecules, simulating potential drug interactions. These residues represent energetically favorable binding hotspots, essential for structure‐based drug design. Following the FTmap predictions, CASTpFold analysis was performed to predict potential ligand‐binding cavities in the accD protein (Figure 11).

The analysis revealed a prominent cavity in the accD protein, with well‐defined boundaries and sufficient volume to accommodate small‐molecule ligands (Figure 11).

**Figure 12:**
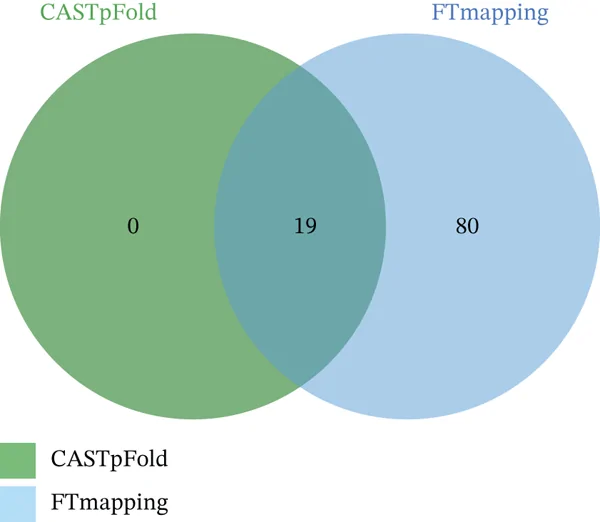
Venn diagram showing the overlap between binding‐site residues predicted by FTMap and CASTpFold.

To ensure the accuracy of binding site prediction, results from CASTpFold and FTMap were compared with identify overlapping residues, which are more likely to represent true ligand‐binding hotspots as shown in Figure 12.

Figure 12 shows the binding site residues predicted by both FTMap and CASTpFold, indicating a common region that is likely involved in ligand binding. The overlapping residues include ARG54, ARG94, PHE127, MET130, GLY131, SER133, GLY163, GLY164, ALA165, ARG166, MET167, GLN168, PRO201, MET203, LEU224, GLY229, PRO230, ARG231, and VAL232.

### 3.10. Molecular Docking Using Discovery Studio

To evaluate the binding affinity and repurposing potential of FDA‐approved drugs against the prioritized target protein accD, molecular docking was performed using the Lib‐Dock algorithm in BIOVIA Discovery Studio. A curated library of FDA‐approved compounds was screened, and the top‐ranked ligands were shortlisted based on a combination of Lib‐Dock scores, hydrogen bonding interactions, nonbonded contacts, and binding free energy values. This multiparametric selection approach ensured prioritization of ligands with both high affinity and stable interactions within the predicted binding pocket of accD.

Previous *in silico* studies targeting *K. pneumoniae* have applied similar docking‐based approaches but were restricted to single‐strain models or nonessential targets, such as murG (involved in peptidoglycan biosynthesis) and lipopolysaccharide biosynthesis enzymes, yielding candidate ligands with theoretical inhibitory potential [59, 60]. However, these studies were limited in translational applicability as their selected targets were not universally conserved across MDR lineages and lacked cross‐validation of binding site predictions.

Table 4 presents the results of structure‐based molecular docking between the accD protein and top 14 shortlisted FDA‐approved compounds based on their Lib‐Dock scores and binding energies, reflecting the stability and strength of their interactions with the active site amino acid residues of accD.

**Table 4:** Fourteen top‐ranked ligands obtained from molecular docking.

Ligand	Lib‐Dock score	Binding energy (kcal/mol)	DrugBank Id	Drug status	Primary clinical use	Reported against *K. pneumoniae*	Antimicrobial activity
Tenapanor	265.09	−112.126	DB11761	Approved/investigational	Irritable bowel syndrome with constipation (IBS‐C).	No	No
Colistin	175.918	−180.667	DB00803	Approved	Bacterial infections caused by susceptible gram‐negative bacteria.	Yes	Yes
POPG	175.015	−96.584	DB11331	Approved/experimental	Infant respiratory distress syndrome (RDS) and used as an emulsifier in pharmaceutical applications.	Reported in studies	Reported in studies
Deferoxamine	168.005	−30.5549	DB00746	Approved/investigational	To treat iron or aluminum toxicity and some blood transfusion dependent anemia.	Reported in studies	Reported in studies
Polymyxin‐B	166.215	−166.58	DB00781	Approved/vet‐approved	Used as antibiotic to treat a wide variety of infections in the body.	Yes	Yes
Copper_oxodotreotide_Cu‐64	162.571	4.4767	DB15873	Approved	Used to detect somatostatin receptors which are highly expressed on neuroendocrine tumors.	No	No
Lypressin	158.319	−72.3589	DB14642	Approved	Used in the treatment of diabetes insipidus.	No	No
Cobicistat	157.338	−30.1189	DB09065	Approved	Increases the effects of atazanavir or darunavir for the treatment of human immunodeficiency virus (HIV) infection.	No	No
Chloramphenicol_palmitate	154.395	−57.2266	DB14658	Approved/vet‐approved	Eye infections, bacterial infections of the outer ear, *Haemophilus influenza*.	Yes	Yes
Viomycin	153.582	−96.184	DB06827	Approved	Primarily used as an antitubercular agent, particularly in cases of multidrug‐resistant tuberculosis (MDR‐TB).	No	Yes
Micafungin	153.003	−96.9621	DB01141	Approved/investigational	Used to treat fungal infections.	No	No
Orlistat	151.457	−70.7568	DB01083	Approved/investigational	Is used to aid weight loss in obese individuals.	No	No
Colfosceril‐palmitate	151.101	−84.8651	DB09114	Approved/investigational/withdrawn	Respiratory distress syndrome (RDS) in premature infants.	No	No
Lymecycline	149.89	−39.3922	DB00256	Approved/investigational	Used for treating moderate to severe acne vulgaris.	Limited reports	Yes

A total of 14 drug candidates were shortlisted based on their highest Lib‐Dock scores to prioritize high affinity interaction and favorable binding energies against the accD protein target. Among these, colistin, polymyxin B, and chloramphenicol palmitate already in clinical use against *K. pneumoniae* served to validate the reliability and accuracy of the docking results. However, the utility of colistin and polymyxin B is increasingly compromised by the rising incidence of resistance: a recent global meta‐analysis estimated the prevalence of colistin resistance among *K. pneumoniae* isolates to be approximately 13% (2010–2023), with increases from ~6% in 2010 to ~18% in 2023 [61]. Polymyxin resistance mechanisms, for example via mutations in the mgrB, PhoPQ, and PmrAB systems have been documented extensively in clinical *K. pneumoniae* strains, particularly among carbapenem‐resistant lineages [62]. In contrast, chloramphenicol, although not a first‐line therapy, has shown promise in the treatment of severe or extensively drug‐resistant (XDR) infections, especially when used in combination with polymyxin B. Such combinations have been reported to exert synergistic effects and induce metabolic perturbations in bacteria that enhance treatment efficacy [63].

Nevertheless, its clinical utility remains constrained by both its well‐documented risk of bone marrow toxicity and the emergence of resistance mechanisms, including enzymatic inactivation via chloramphenicol acetyltransferase and efflux‐mediated resistance [64]. Copper oxodotreotide Cu64 was excluded due to its positive binding energy, indicating an unstable interaction. Lymecycline, although belonging to the tetracycline class, has limited published data in *K. pneumoniae*, and its efficacy appears weak or inconsistent in available studies; thus, it was not prioritized. Two other compounds, deferoxamine and 1‐palmitoyl‐2‐oleoyl‐sn‐glycero‐3‐(phospho‐rac‐(1‐glycerol)) (POPG), have known antimicrobial or membrane‐modulatory properties, but have not been demonstrated in the literature to treat *K. pneumoniae* [65, 66].

This preliminary screening resulted in nine candidate ligands, 1‐palmitoyl‐2‐oleoyl‐sn‐glycero‐3‐(phospho‐rac‐(1‐glycerol)), cobicistat, colfosceril_palmitate, deferoxamine, lypressin, orlistat, tenapanor, viomycin, and micafungin, which were subsequently subjected to detailed interaction analysis.

To evaluate the binding characteristics of the shortlisted compounds, a detailed interaction analysis was performed focusing on H‐bonded and nonbonded contacts with the active site residues of the accD protein (Figure S2 and Table S3) summarizes the H‐bonded and nonbonded interaction profiles of the nine shortlisted FDA‐approved drug candidates docked against the accD protein. The analysis indicates that most ligands formed multiple hydrogen bonds with key active site residues, enhancing binding affinity and stability within the binding pocket. Notably, tenapanor, deferoxamine, micafungin, viomycin, and lypressin exhibited extensive hydrogen bonding along with significant nonbonded interactions, suggesting their potential as repurposable drug candidates. In contrast, orlistat formed only a single hydrogen bond, suggesting comparatively weaker polar interactions; although it retained additional nonbonded contacts, its limited hydrogen bonding likely reduces specificity and stability, and it was therefore excluded from further consideration.

### 3.11. MD Simulations

MD simulations were performed for the accD protein in complex with the seven top‐ranked FDA‐approved compounds that showed strong binding affinity in the docking studies. Here, the native protein was used as a reference. The analysis was carried out to examine the protein stability, structural changes, compactness, flexibility as well as key interactions in the docking analysis. This investigation focused on key factors, such as RMSD, RMSF, Rg, and hydrogen‐bond interaction.

### 3.12. RMSD Analysis

**Figure 13:**
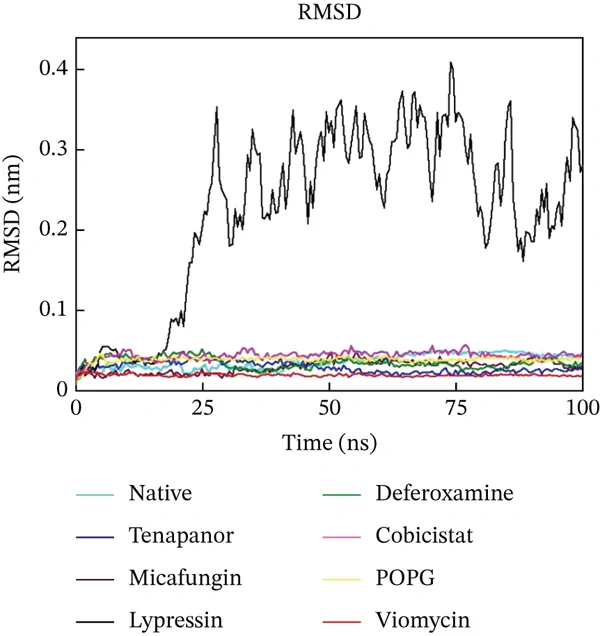
RMSD profiles of native accD and accD‐ligand complexes during the 100 ns molecular dynamics simulation.

The RMSD plot is essential for assessing the equilibration and stability of protein structures in which smaller RMSD values indicate greater stability [67]. The RMSD analysis of accD protein in the presence of seven different drugs against MDR pathogens was analyzed and shown in Figure 13. This study was done to see the overall structural deviation of the accD protein‐drug complexes from their initial conformation throughout the 100 ns simulation.

Figure 13 shows that accD in its native form and in complex with almost all the compounds, except for lypressin, exhibited low fluctuations (0.01–0.06 nm) during the simulation. accD in complex with the other six compounds displayed native‐like fluctuation patterns, indicating stable ligand–protein interactions and good structural stability.

Among them, lypressin (black) showed larger variations in their RMSD fluctuations, ranging from 0.02 to 0.42 nm, particularly after 15 ns, indicating that the accD protein might not be stable in the presence of this compound. This analysis suggests poor ligand–protein interactions and lower structural stability. Here, accD in presence of viomycin performed the best result, suggesting the lowest fluctuations among all tested compounds. The protein in presence of tenapanor, micafungin, cobicistat, deferoxamine, and POPG also showed relatively low fluctuations often comparable to, and in some cases better than the native whereas lypressin being more unstable.

Thus, the structural stability trend based on RMSD was: viomycin > tenapanor > deferoxamine > micafungin > POPG > native > cobicistat > lypressin.

### 3.13. RMSF Analysis

**Figure 14:**
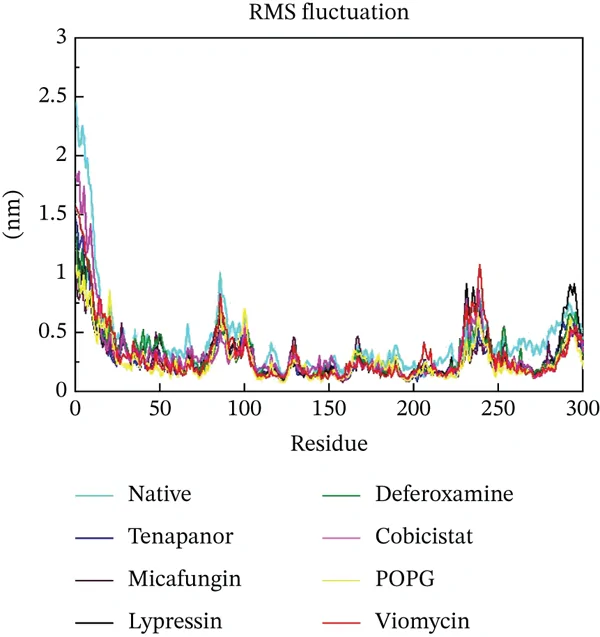
RMSF analysis of native accD and accD–ligand complexes during the 100 ns molecular dynamics simulation.

Residue‐wise fluctuations were performed using RMSF calculations, which offer insight into the flexibility of protein residues during simulation. Higher RMSF values indicate greater flexibility, whereas lower values suggest restricted movements [68]. Figure 14 illustrates the RMSF analysis of amino acid residues for the native accD protein and its complexes with seven different drugs during the course of 100 ns simulations. Most residues exhibited minimal fluctuations, indicating structural rigidity, particularly around the active site.

From Figure 14, we can see that the RMSF profiles of the protein‐drug complexes remained mostly between 0.2–1.5 nm. Among all these compounds, accD in complex with tenapanor, micafungin, deferoxamine, and POPG exhibited the lowest fluctuations, ranging from approximately 0.20–0.75 nm. Notably, four complexes exhibited much lower fluctuations than the native (cyan) structure in the residue region 110–225, with values ranging from 0.20 to 0.50 nm, indicating high backbone stability of accD and reduced local flexibility. In contrast, the accD‐Lypressin complex showed moderate fluctuations, with peaks of approximately 0.24–1.00 nm.

In presence of cobicistat and viomycin, accD displayed slightly higher fluctuations with peaks reaching approximately 1.6–1.85 nm, but the fluctuations that were observed mostly at the beginning up to 15 residues. After 15 residues, these two became stable and showed moderate fluctuations. Thus, the structural stability trend based on RMSF was as follows: tenapanor > deferoxamine > cobicistat > micafungin > POPG > lypressin > viomycin > native.

### 3.14. Rg Analysis

**Figure 15:**
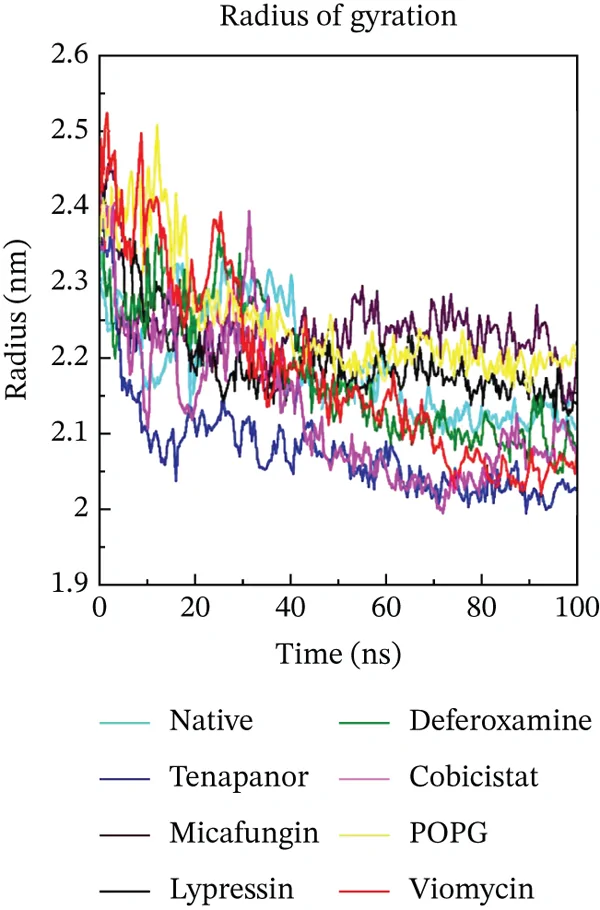
Radius of gyration (Rg) analysis of native accD and accD–ligand complexes during the 100 ns molecular dynamics simulation.

The Rg was used to check how compact the protein remained during the simulation [4]. The Rg values (as shown in Figure 15) stayed steady within a narrow range indicating the protein stayed compact and well folded without major structural changes.

The figure demonstrates that all of the systems had Rg values between 2.3 and 2.52 nm, which were followed by a gradual decline and stabilization after about 20–30 ns. This indicates proper equilibration without global unfolding, with the native protein remaining compact at an average Rg slightly above 2.1 nm, suggesting inherent structural stability throughout the simulation period.

Among the compounds, the tenapanor and cobicistat complexes showed the most compact trajectories approximately after 40 ns with stabilized Rg values equal to or marginally lower than the native state. This means that when accD bound to these compounds it suggested stabilization of the protein′s tertiary structure. accD in complex with viomycin, deferoxamine, micafungin, lypressin as well as in its native form maintained consistent Rg profiles. These molecules preserve the overall fold but may induce a modest increase in structural flexibility.

However, POPG, and viomycin showed the greatest fluctuations at the beginning of the simulation before stabilizing after 20 ns, demonstrating that the protein–ligand combination had minimal structural flexibility. These findings indicate that the following structural stability order based on Rg values: tenapanor > cobicistat > deferoxamine > viomycin > native > micafungin > lypressin > POPG.

### 3.15. H‐Bond Interaction Analysis

**Figure 16:**
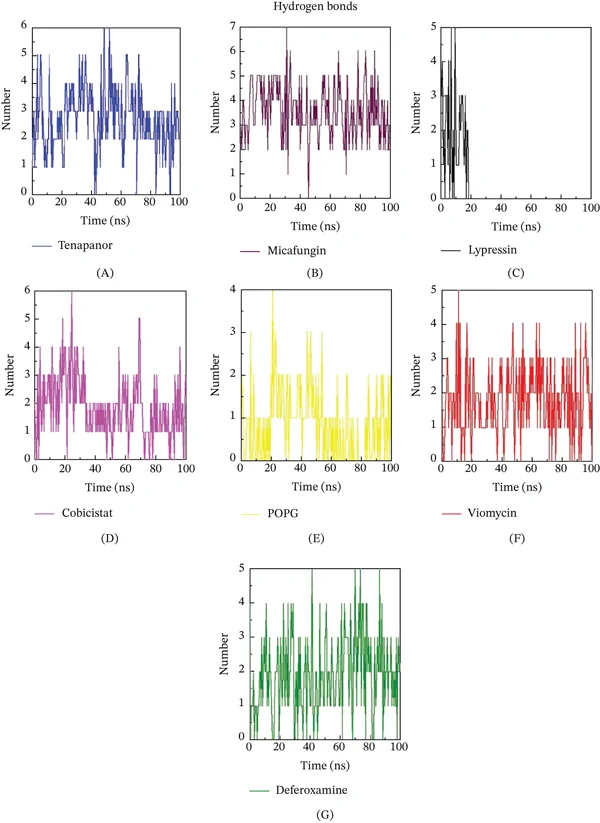
Hydrogen‐bond occupancy profiles of seven accD–ligand complexes during the 100 ns molecular dynamics simulation. (A) Tenapanor, (B) micafungin, (C) lypressin, (D) cobicistat, (E) POPG, (F) viomycin, and (G) deferoxamine.

Nonbonded interactions, mainly hydrogen bonds, play a crucial role in stabilizing protein‐ligand complexes [69]. Thus, the number of hydrogen bonds formed between the ligand and the protein was analyzed over the period of simulation (as shown in Figure 16).

The H‐bond analysis displayed that six compounds maintained a good number of hydrogen bonds during the simulation. Micafungin and tenapanor formed the most stable order and maintained up to five hydrogen bonds, occasionally reaching up to six to seven. cobicistat displayed the next best pattern, with four bonds throughout, sometimes reaching up to six. Deferoxamine and viomycin also showed favorable bonding and maintained up to four bonds, sometimes reaching up to five. POPG showed steady hydrogen bonding that spikes up to two, sometimes reaching up to four.

Lypressin did form hydrogen bonds up to four to five in the early phase of the simulation that is observed only during the first 20 ns, suggesting transient hydrogen‐bonding interactions that disappear in the later part of the trajectory. It also indicates an unstable or weakly persistent interaction. Based on hydrogen‐bond stability, the order of structural stability is as follows: micafungin > tenapanor > cobicistat > deferoxamine > viomycin > POPG > lypressin.

To further validate the hydrogen‐bonding behavior observed during the simulations, a refined hydrogen‐bond occupancy and lifetime analysis was performed using the extended MD trajectory (Table 5) [70].

**Table 5:** Comparative hydrogen‐bonding characteristics of the protein‐ligand complexes during 100 ns molecular dynamics simulations.

Ligand	Simulation Time (ns)	Total H‐bond pairs	Average H‐bonds	Maximum occupancy (%)	Dominant hydrogen bond
Cobicistat	100	11	1.821	70.21	ARG262 NH2 → LIG N32
Deferoxamine	100	17	1.881	18.23	LIG O10 → GLU265 N
Lypressin	100	3	0.437	9.81	LIG N24 → ASP50 OD1
Micafungin	100	15	3.542	92.81	GLY244 N → LIG N70
Palmitoyl‐POPG	100	9	0.939	13.79	ARG247 NH1 → POPG O31
Tenapanor	100	97	2.707	71.14	ARG247 NH2 → LIG O34
Viomycin	100	102	4.464	67.88	ARG60 NH1 → LIG O13

Hydrogen bond analysis was performed over the 100 ns MD trajectories to evaluate the stability and persistence of protein–ligand interactions (Table 5 and Figures S3, S4, S5, S6, S7, S8, and S9). Considerable differences were observed among the investigated ligands with respect to the number of hydrogen‐bond pairs, average hydrogen‐bond occupancy, and the persistence of individual donor–acceptor interactions.

Among the investigated ligands, viomycin exhibited the most extensive hydrogen‐bonding network, forming 102 unique donor–acceptor pairs and maintaining the highest average number of hydrogen bonds throughout the simulation (4.464). Although its strongest interaction (ARG60 NH1 → LIG O13) displayed a maximum occupancy of 67.88%, the large number of simultaneous hydrogen bonds indicates that ligand stabilization is achieved through a distributed interaction network rather than a single dominant contact.

Tenapanor also established an extensive hydrogen‐bonding network consisting of 97 unique donor–acceptor pairs, with an average of 2.707 hydrogen bonds during the simulation. Its most persistent interaction, ARG247 NH2 → LIG O34, remained occupied for 71.14% of the trajectory, together with several additional interactions exhibiting occupancies greater than 30%, suggesting stable anchoring within the active‐site binding pocket.

In contrast, micafungin formed only 15 unique hydrogen‐bond pairs but displayed the highest individual hydrogen‐bond occupancy (92.81%) among all complexes. The GLY244 N → LIG N70 interaction remained nearly continuous throughout the simulation, indicating exceptionally strong stabilization through a limited number of highly persistent hydrogen bonds. Furthermore, micafungin maintained an average of 3.542 hydrogen bonds, demonstrating that its binding is supported by both high interaction persistence and multiple concurrent hydrogen bonds.

Cobicistat formed 11 unique hydrogen‐bond pairs with an average of 1.821 hydrogen bonds during the simulation. Despite the relatively small interaction network, the highly persistent ARG262 NH2 → LIG N32 hydrogen bond exhibited an occupancy of 70.21%, highlighting the importance of a few strong interactions in stabilizing the complex.

The remaining ligands exhibited comparatively weaker hydrogen‐bonding behavior. Deferoxamine generated 17 hydrogen‐bond pairs with an average of 1.881 hydrogen bonds, although its strongest interaction (LIG O10 → GLU265 N) reached only 18.23% occupancy. Palmitoyl‐POPG formed nine hydrogen‐bond pairs with an average of 0.939 hydrogen bonds, dominated by the ARG247 NH1 → POPG O31 interaction (13.79% occupancy). Lypressin showed the least persistent hydrogen‐bonding pattern, forming only three hydrogen‐bond pairs with an average of 0.437 hydrogen bonds, and its strongest interaction (LIG N24 → ASP50 OD1) exhibited a maximum occupancy of 9.81%.

Micafungin achieved the highest interaction persistence through a small number of exceptionally stable hydrogen bonds, whereas viomycin and tenapanor stabilized the complex through extensive hydrogen‐bonding networks involving numerous donor–acceptor pairs. Cobicistat also demonstrated strong binding despite forming relatively few hydrogen bonds, indicating that interaction persistence rather than interaction number can play a dominant role in complex stabilization. Conversely, deferoxamine, palmitoyl‐POPG, and lypressin displayed weaker hydrogen‐bond persistence and lower average hydrogen‐bond numbers, suggesting comparatively reduced contributions of hydrogen bonding to overall complex stability.

### 3.16. PCA‐Based FEL Analysis

PCA was performed to investigate the dominant collective motions of the native accD protein and its complexes with the selected FDA‐approved compounds. The eigenvalue distributions demonstrated that the first principal component (PC1) exhibited the highest eigenvalue in all systems, followed by PC2, whereas the remaining principal components showed a gradual decline in their contributions. This indicates that the essential conformational dynamics of both the native protein and ligand‐bound complexes were predominantly captured by the first two principal components, justifying their selection as reaction coordinates for subsequent PCA‐based FEL analysis.

The native accD protein exhibited the highest variance along the first principal component (PC1 eigenvalue = 193.39), suggesting that, relative to the ligand‐bound complexes, the apo protein sampled larger‐amplitude collective conformational motions during the simulation. Among the ligand‐bound complexes, cobicistat displayed the highest PC1 eigenvalue (84.30), followed by viomycin (61.04), lypressin (50.06), deferoxamine (47.36), tenapanor (45.27), POPG (26.24), and micafungin (19.24). The comparatively lower PC1 values observed for several ligand‐bound systems suggest that ligand binding generally restricted the large‐scale collective motions of accD to varying extents. Although the magnitude of collective motion differed among the complexes, all systems exhibited a characteristic decrease in eigenvalues after the first two principal components, indicating that the dominant conformational dynamics were adequately represented by PC1 and PC2.

**Figure 17:**
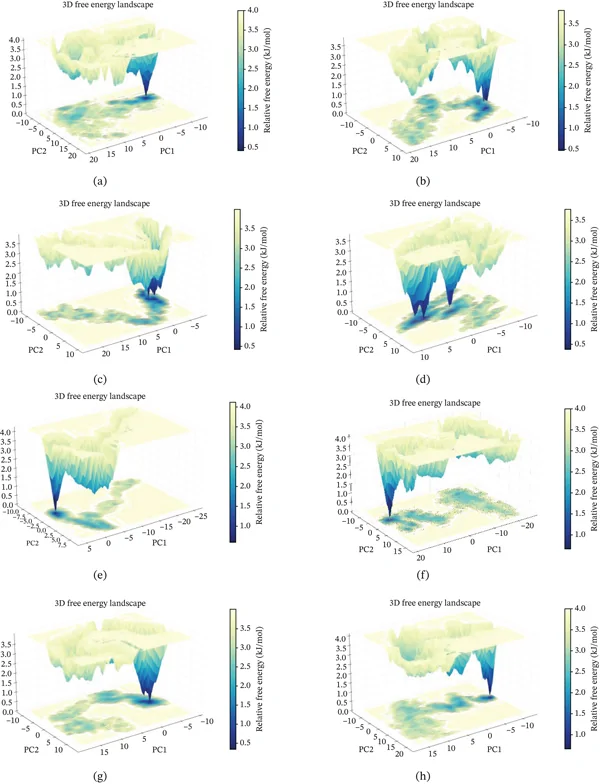
Principal component analysis (PCA)‐based free energy landscapes (FELs) of the native accD protein and ligand‐bound complexes generated from 100 ns molecular dynamics simulations. (a) Cobicistat, (b) deferoxamine, (c) lypressin, (d) micafungin, (e) native accD, (f) 1‐palmitoyl‐2‐oleoyl‐sn‐glycero‐3‐(phospho‐rac‐(1‐glycerol)) (POPG), (g) tenapanor, and (h) viomycin.

PCA‐based FEL analysis was performed using the first two principal components (PC1 and PC2) to investigate the thermodynamic stability and conformational dynamics of the native accD protein and its complexes throughout the 100 ns MD simulations. The two‐dimensional (2D) PCA‐based FELs of the native accD protein and all ligand‐bound complexes are provided in the Supporting Information (Figure S10). To further examine the conformational energy landscape, 3D PCA‐based FELs were generated for the native accD protein and all ligand‐bound complexes (Figure 17).

The 3D energy surfaces depict the relative Gibbs free energy of the sampled conformational states. Dark blue regions represent low‐energy minima corresponding to thermodynamically favorable and stable conformations, whereas lighter green to yellow regions indicate higher‐energy and less populated conformational states. The compactness and distribution of the low‐energy basins reflect differences in the conformational stability and dynamic behavior of the respective protein‐ligand complexes.

The low‐energy basins observed in the FELs correspond to thermodynamically favorable conformational states, whereas broader and more dispersed energy distributions indicate increased conformational flexibility and transitions between metastable states.

Among the investigated systems, the tenapanor and micafungin complexes exhibited the most compact and well‐defined low‐energy conformational basins. Tenapanor displayed a pronounced funnel‐shaped energy minimum with a highly localized conformational distribution, suggesting strong thermodynamic confinement and limited structural transitions during the simulation. Similarly, micafungin showed distinct deep energy minima connected by narrow transition pathways, indicating stable sampling of energetically favorable conformations while maintaining limited conformational flexibility. These observations are consistent with their persistent hydrogen‐bond interactions and stable MD trajectories.

The cobicistat and viomycin complexes also occupied well‐defined low‐energy regions, although their energy landscapes were comparatively broader than those observed for tenapanor and micafungin. Cobicistat exhibited multiple interconnected low‐energy basins, suggesting the presence of several energetically favorable conformational states with moderate structural adaptability. Likewise, viomycin displayed a dominant energy minimum accompanied by adjacent low‐energy conformations, indicating stable binding while allowing limited conformational transitions throughout the simulation.

In contrast, the deferoxamine, POPG, and lypressin complexes explored relatively broader conformational spaces characterized by multiple shallow energy minima and wider transition regions. Deferoxamine exhibited several accessible low‐energy conformations connected through intermediate states, indicating moderate conformational flexibility. POPG displayed dispersed low‐energy regions extending over a larger conformational space, reflecting increased structural heterogeneity during the simulation. Similarly, lypressin demonstrated an elongated free‐energy distribution with several partially connected minima, suggesting continuous transitions among multiple metastable conformations and comparatively reduced thermodynamic confinement.

The native accD protein exhibited the broadest conformational landscape with multiple distinct low‐energy basins distributed across a wider region of the principal component space, reflecting its intrinsic flexibility in the absence of ligand binding. In comparison, ligand binding generally restricted the conformational space sampled by accD, particularly in the tenapanor, micafungin, cobicistat, and viomycin complexes, indicating enhanced structural stabilization upon ligand association.

Based on the compactness of the low‐energy basins, conformational confinement, and persistence of energetically favorable states, the thermodynamic stability of the investigated systems can be broadly ranked as follows: tenapanor ≈ micafungin > cobicistat ≈ viomycin > deferoxamine > POPG > lypressin > native.

Overall, the integrated MD analyses consistently demonstrated that ligand binding significantly influenced the conformational stability and dynamic behavior of accD. Although conventional MD descriptors (RMSD, RMSF, and Rg) established the structural stability of the protein‐ligand complexes, hydrogen‐bond occupancy analysis provided detailed insights into the persistence of intermolecular interactions responsible for complex stabilization. PCA revealed that the dominant collective motions of the systems were primarily represented by the first two principal components, and the subsequent PCA‐based FEL analysis further confirmed the thermodynamic stability of the ligand‐bound complexes by identifying well‐defined low‐energy conformational states. Among the investigated compounds, tenapanor, micafungin, cobicistat, and deferoxamine generally exhibited favorable structural and thermodynamic characteristics across multiple complementary analyses. Collectively, these findings suggest that accD may represent a conserved therapeutic target in MDR *K. pneumoniae* and identify these FDA‐approved compounds as potential candidates for further experimental validation.

## 4. Discussion

The increasing prevalence of MDR *K. pneumoniae* has become a major public health concern as resistance to multiple antibiotics has reduced the effectiveness of available treatment options [3]. Computational target discovery has become a practical early‐stage approach for antimicrobial research because subtractive and comparative genomics can prioritize essential, pathogen‐specific, and non‐host‐homologous proteins before labor‐intensive experimental validation [71, 72]. Such pipelines commonly integrate genome comparison, essentiality screening, subcellular localization, pathway analysis, structural modeling, and docking‐based prioritization to reduce large proteomes to a smaller set of biologically relevant candidates [73, 74] .

A key strength of this type of workflow is its multilayered filtering strategy, in which conserved proteins are progressively refined by host nonhomology, essentiality, druggability, and functional relevance, thereby improving confidence in shortlisted targets [14, 40]. Across several pathogens, this strategy has repeatedly reduced thousands of proteins to a limited number of candidate targets suitable for downstream structural and pharmacological analysis [75, 76]. Prioritizing cytoplasmic proteins that are essential for bacterial survival and lack close similarity to host proteins is especially attractive because it tends to minimize off‐target toxicity while preserving therapeutic relevance [72].

Among plausible candidates, accD/acetyl‐CoA carboxylase is biologically compelling because bacterial ACC catalyzes the first committed step of fatty acid biosynthesis, a pathway required for membrane biogenesis and cellular viability [77, 78]. ACC converts acetyl‐CoA to malonyl‐CoA through coordinated biotin carboxylase and carboxyltransferase activities, placing accD at the center of lipid precursor generation [78, 79]. Bacterial ACC has long been regarded as an attractive antibacterial target, and both genetic and chemical studies support its vulnerability, including the discovery of potent ACC inhibitors with antibacterial activity and resistance mutations mapping directly to accD subunits [76, 80]. Selective inhibition is also plausible because bacterial and eukaryotic ACC systems differ sufficiently to permit host‐sparing targeting [77, 78].

Structure‐based screening and drug repurposing are logical downstream extensions once a target has been prioritized, particularly when crystal structures are unavailable and modeled proteins can support docking workflows [75, 81, 40, 82]. Repurposing approved compounds is especially attractive because it can reduce development time, cost, and safety uncertainty relative to de novo antibiotic discovery [71, 82]. Similar *K. pneumoniae*‐focused *in silico* studies have already used FDA‐approved libraries, docking, and MD simulations to identify candidate inhibitors against resistant bacterial enzymes, illustrating the feasibility of this strategy in the same pathogen [82, 83].

Molecular docking alone provides a static interaction snapshot, so MD simulations are routinely used to evaluate whether protein‐ligand complexes remain stable under dynamic conditions [75, 84, 85]. Common descriptors such as RMSD, RMSF, Rg, hydrogen‐bonding patterns, and free‐energy analyses help determine whether ligand binding stabilizes the target and restricts conformational flexibility [85, 86]. More broadly, repurposed drugs can show unexpected antibacterial activity or synergistic effects against gram‐negative pathogens, including MDR *K. pneumoniae*, even when they were originally developed for nonantibacterial indications [71, 87].

These findings support viewing the present results as a prioritization framework rather than definitive proof of efficacy, because fully computational studies still require experimental confirmation of target essentiality, antimicrobial activity, uptake, pharmacodynamics, and resistance liability [71, 75, 84]. Accordingly, gene‐level validation, susceptibility testing, mechanistic assays, and *in vivo* studies remain necessary before candidate target–drug combinations can be advanced toward preclinical development [14, 40, 88].

## 5. Conclusion

The increasing prevalence of MDR *K. pneumoniae* poses a major challenge to current antimicrobial therapy and highlights the need for alternative therapeutic strategies. In this study, an integrated computational workflow combining comparative and subtractive proteomics with structure‐based drug repurposing was employed to identify conserved, essential, and non‐homologous bacterial targets. By prioritizing pathogen‐specific proteins such as accD, this approach may help reduce the risk of host toxicity while improving the likelihood of identifying broad‐spectrum therapeutic candidates. Furthermore, the application of virtual screening, molecular docking, and MD simulations with FDA‐approved drugs demonstrates the utility of computational methods for the early‐stage identification and evaluation of potential drug candidates.

Despite these promising findings, the results are based on computational predictions and therefore require experimental validation through *in vitro* and *in vivo* studies to confirm target essentiality, binding affinity, and therapeutic efficacy. Overall, this study provides a systematic framework for the identification of potential drug targets and repurposable compounds and offers a basis for future experimental studies aimed at developing new therapeutic strategies against MDR *K. pneumoniae*.

In conclusion, this study identified accD as a conserved and potentially therapeutic target across multiple MDR *K. pneumoniae* strains and highlighted several FDA‐approved compounds as promising candidates for drug repurposing. Although the computational findings provide preliminary evidence supporting the feasibility of this approach, experimental validation will be necessary to confirm target essentiality, antimicrobial activity, and therapeutic efficacy. Nevertheless, the presented workflow offers a useful framework for accelerating antimicrobial target discovery and drug repurposing against MDR bacterial pathogens.

## Funding

This study was supported by the Department of Science and Technology, Ministry of Science and Technology, India (10.13039/501100001409), DST PURSE Project (No. SR/PURSE/2022/143); Department of Health Research, India (10.13039/501100009104, F. No. R.12012/10/2024‐HR/E‐Office:8310878).

## Disclosure

The authors reviewed and verified all content and take full responsibility for the final version.

## Ethics Statement

The authors have nothing to report.

## Conflicts of Interest

The authors no conflicts of interest.

## Supporting information


**Supporting Information** Additional supporting information can be found online in the Supporting Information section. Table S1 presents the genomic features and virulence‐associated annotations of the six *K. pneumoniae* strains based on RAST analysis. Table S2 provides the GO enrichment analysis, including biological process, cellular component, and MF categories. Table S3 summarizes the molecular interactions of the shortlisted ligands with the accD protein. Figures S1 and S2 present the RAST subsystem distribution and two‐dimensional ligand–accD interaction diagrams, respectively. Figures S3, S4, S5, S6, S7, S8, and S9 show hydrogen‐bond occupancy analyses of the accD–ligand complexes during 100 ns molecular dynamics simulations, whereas Figure S10 presents PCA‐based 2D free‐energy landscapes of the native accD protein and ligand‐bound complexes.

## Data Availability

Data sharing is not applicable to this article as no datasets were generated or analyzed during the current study.
