# Supplementary material for: Comparative and Subtractive Genomics Analysis of Multidrug‐Resistant Klebsiella pneumoniae Strains for Novel Target Identification and Drug Repurposing Strategies
Source: Int J Microbiol. 2026 Sep 6;2026:1460586. doi: 10.1155/ijm/1460586 (PMC13547958; doi:10.1155/ijm/1460586)
Supplement: Supplementary file 1 [file IJM-2026-1460586-s001.docx]

**Comparative and Subtractive Genomics Analysis of Multidrug-Resistant *Klebsiella pneumoniae* Strains for Novel Target Identification and Drug Repurposing Strategies**

Manasi Bora,^1^ Prokriti Paul,^1^ Sanchaita Rajkhowa,^1*^ Harshita Sonar,^1^ Borakha Bura Gohain,^1^ Magdi E. A. Zaki,^2*^ and Dhamodharan Prabhu^3^

^1^Centre for Biotechnology and Bioinformatics, Dibrugarh University, Dibrugarh, Assam-786004, India.

^2^Department of Chemistry, Imam Mohammad Ibn Saud Islamic University (IMSIU), Riyadh, Saudia Arabia.

^3^Centre for Drug Discovery, Department of Biotechnology, Karpagam Academy of Higher Education, Coimbatore, Tamil Nadu, India

^*^Corresponding author (s): (Dr. Sanchaita Rajkhowa) s_rajkhowa@dibru.ac.in (ORCID ID: 0000-0002-4834-2654) and (Dr. Magdi E. A. Zaki) mezaki@imamu.edu.sa

**SI Table 1:** Genomic features and virulence-associated annotations of six *Klebsiella pneumoniae* strains based on RAST analysis.

| Strain name | VA585-22 | ST231 | ST16 | ST307 | ST11 | HS11286 |
| --- | --- | --- | --- | --- | --- | --- |
| No of contigs | 1 | 1 | 1 | 1 | 1 | 1 |
| Size (bp) | 5334759 | 5450665 | 5324293 | 5365823 | 5210054 | 5333942 |
| No of Sub systems | 555 | 555 | 556 | 557 | 538 | 588 |
| No of Coding Sequences | 4967 | 5157 | 5031 | 5054 | 4967 | 5190 |
| No of RNAs | 111 | 112 | 116 | 113 | 108 | 112 |
| No of genes encoded for virulence disease and defence | 92 | 92 | 92 | 92 | 91 | 102 |
| No of genes for adhesion | 6 | 6 | 6 | 6 | 6 | 6 |
| No of genes for Antibiotic Resistance | 70 | 70 | 70 | 70 | 69 | 72 |
| No of genes for Antibacterial Peptides | 4 | 4 | 4 | 4 | 4 | 12 |
| No of genes for Invasion and intracellular resistance | 12 | 12 | 12 | 12 | 12 | 12 |

**SI Table 2(A):** GO biological process

| **GO biological process complete** | ***E.coli*- REF**  **LIST (4403)** | **Upload_1 (3)** | **Upload_1**  **(expected)** | **upload_1**  **(over/under)** | **upload_1 (fold**  **Enrichment)** | **upload_1**  **(raw P-value)** |
| --- | --- | --- | --- | --- | --- | --- |
| negative regulation of macromolecule biosynthetic process (GO:0010558) | 199 | 3 | 0.14 | + | 22.13 | 9.10E-05 |
| negative regulation of biosynthetic process (GO:0009890) | 200 | 3 | 0.14 | + | 22.02 | 9.24E-05 |
| negative regulation of macromolecule metabolic process (GO:0010605) | 215 | 3 | 0.15 | + | 20.48 | 1.15E-04 |
| negative regulation of metabolic process (GO:0009892) | 217 | 3 | 0.15 | + | 20.29 | 1.18E-04 |
| negative regulation of cellular process (GO:0048523) | 257 | 3 | 0.18 | + | 17.13 | 1.97E-04 |
| negative regulation of biological process (GO:0048519) | 259 | 3 | 0.18 | + | 17 | 2.01E-04 |
| regulation of gene expression (GO:0010468) | 463 | 3 | 0.32 | + | 9.51 | 1.16E-03 |
| regulation of macromolecule biosynthetic process (GO:0010556) | 465 | 3 | 0.32 | + | 9.47 | 1.17E-03 |
| regulation of biosynthetic process (GO:0009889) | 471 | 3 | 0.32 | + | 9.35 | 1.22E-03 |
| regulation of primary metabolic process (GO:0080090) | 481 | 3 | 0.33 | + | 9.15 | 1.30E-03 |
| regulation of cell-substrate adhesion (GO:0010810) | 2 | 1.00E  +00 | 0 | + | > 100 | 1.36E-03 |
| regulation of cell adhesion (GO:0030155) | 2 | 1.00E  +00 | 0 | + | > 100 | 1.36E-03 |
| long-chain fatty acid biosynthetic process (GO:0042759) | 2 | 1.00E  +00 | 0 | + | > 100 | 1.36E-03 |
| regulation of macromolecule metabolic process (GO:0060255) | 494 | 3 | 0.34 | + | 8.91 | 1.40E-03 |
| regulation of metabolic process (GO:0019222) | 505 | 3 | 0.34 | + | 8.72 | 1.50E-03 |
| long-chain fatty acid metabolic process (GO:0001676) | 4 | 1.00E  +00 | 0 | + | > 100 | 2.72E-03 |
| malonyl-CoA biosynthetic process (GO:2001295) | 4 | 1.00E  +00 | 0 | + | > 100 | 2.72E-03 |
| malonyl-CoA metabolic process (GO:2001293) | 4 | 1.00E  +00 | 0 | + | > 100 | 2.72E-03 |
| regulation of cellular process (GO:0050794) | 629 | 3 | 0.43 | + | 7 | 2.90E-03 |
| negative regulation of DNA-templated transcription (GO:0045892) | 150 | 2 | 0.1 | + | 19.57 | 3.38E-03 |
| negative regulation of RNA biosynthetic process (GO:1902679) | 150 | 2 | 0.1 | + | 19.57 | 3.38E-03 |
| negative regulation of RNA metabolic process (GO:0051253) | 154 | 2 | 0.1 | + | 19.06 | 3.56E-03 |
| regulation of biological process (GO:0050789) | 690 | 3 | 0.47 | + | 6.38 | 3.83E-03 |
| negative regulation of nucleobase-containing compound metabolic process (GO:0045934) | 164 | 2 | 0.11 | + | 17.9 | 4.04E-03 |
| biological regulation (GO:0065007) | 724 | 3 | 0.49 | + | 6.08 | 4.43E-03 |
| thioester biosynthetic process (GO:0035384) | 8 | 1.00E  +00 | 0.01 | + | > 100 | 5.44E-03 |
| acyl-CoA biosynthetic process (GO:0071616) | 8 | 1.00E  +00 | 0.01 | + | > 100 | 5.44E-03 |
| negative regulation of DNA-templated transcription initiation (GO:2000143) | 16 | 1 | 0.01 | + | 91.73 | 1.09E-02 |
| thioester metabolic process (GO:0035383) | 17 | 1 | 0.01 | + | 86.33 | 1.15E-02 |
| acyl-CoA metabolic process (GO:0006637) | 17 | 1 | 0.01 | + | 86.33 | 1.15E-02 |
| fatty acid biosynthetic process (GO:0006633) | 25 | 1 | 0.02 | + | 58.71 | 1.69E-02 |
| negative regulation of translation (GO:0017148) | 26 | 1 | 0.02 | + | 56.45 | 1.76E-02 |
| negative regulation of protein metabolic process (GO:0051248) | 31 | 1 | 0.02 | + | 47.34 | 2.10E-02 |
| regulation of RNA biosynthetic process (GO:2001141) | 392 | 2 | 0.27 | + | 7.49 | 2.23E-02 |
| regulation of DNA-templated transcription (GO:0006355) | 392 | 2 | 0.27 | + | 7.49 | 2.23E-02 |
| regulation of RNA metabolic process (GO:0051252) | 402 | 2 | 0.27 | + | 7.3 | 2.34E-02 |
| regulation of nucleobase-containing compound metabolic process (GO:0019219) | 417 | 2 | 0.28 | + | 7.04 | 2.52E-02 |
| amide biosynthetic process (GO:0043604) | 44 | 1 | 0.03 | + | 33.36 | 2.97E-02 |
| regulation of DNA-templated transcription initiation (GO:2000142) | 44 | 1 | 0.03 | + | 33.36 | 2.97E-02 |
| monocarboxylic acid biosynthetic process (GO:0072330) | 48 | 1 | 0.03 | + | 30.58 | 3.24E-02 |
| negative regulation of gene expression (GO:0010629) | 54 | 1 | 0.04 | + | 27.18 | 3.64E-02 |
| regulation of translation (GO:0006417) | 59 | 1 | 0.04 | + | 24.88 | 3.97E-02 |
| cell adhesion (GO:0007155) | 62 | 1 | 0.04 | + | 23.67 | 4.17E-02 |
| post-transcriptional regulation of gene expression (GO:0010608) | 66 | 1 | 0.04 | + | 22.24 | 4.43E-02 |
| sulfur compound biosynthetic process (GO:0044272) | 66 | 1 | 0.04 | + | 22.24 | 4.43E-02 |
| purine-containing compound biosynthetic process (GO:0072522) | 67 | 1 | 0.05 | + | 21.91 | 4.50E-02 |
| fatty acid metabolic process (GO:0006631) | 69 | 1 | 0.05 | + | 21.27 | 4.63E-02 |
| phosphorelay signal transduction system (GO:0000160) | 74 | 1 | 0.05 | + | 19.83 | 4.96E-02 |

**SI Table 2(B):** GO cellular component

| **Cellular component complete** | ***E. coli* – REFLIST**  **(4403)** | **Upload_1(3)** | **upload_1**  **(expected)** | **upload_1**  **(over/under)** | **upload_1**  **(fold Enrichment)** | **upload_1**  **(raw P-value)** |
| --- | --- | --- | --- | --- | --- | --- |
| acetate CoA-transferase complex (GO:0009329) | 2 | 1 | 0 | + | > 100 | 1.36E-03 |
| acetyl-CoA carboxylase complex (GO:0009317) | 4 | 1 | 0 | + | > 100 | 2.72E-03 |
| nucleoid (GO:0009295) | 27 | 1 | 0.02 | + | 54.36 | 1.83E-02 |
| cytoplasm (GO:0005737) | 1475 | 3 | 1 | + | 2.99 | 3.75E-02 |
| intracellular anatomical structure (GO:0005622) | 1507 | 3 | 1.03 | + | 2.92 | 4.00E-02 |

**SI Table 2(C):** GO molecular function

| **GO molecular function complete** | ***E.coli-*REFLIST**  **(4403)** | **Upload_1 .(3)** | **Upload_1**  **(expected)** | **Upload_1**  **(over/under)** | **upload_1**  **(fold Enrichment)** | **upload_1 (raw P-value)** |
| --- | --- | --- | --- | --- | --- | --- |
| DNA binding (GO:0003677) | 535 | 3 | 0.36 | + | 8.23 | 1.79E-03 |
| transcription regulatory region nucleic acid binding (GO:0001067) | 133 | 2 | 0.09 | + | 22.07 | 2.66E-03 |
| transcription cis-regulatory region binding (GO:0000976) | 133 | 2 | 0.09 | + | 22.07 | 2.66E-03 |
| ligase activity, forming carbon-carbon bonds (GO:0016885) | 4 | 1 | 0 | + | > 100 | 2.72E-03 |
| CoA carboxylase activity (GO:0016421) | 4 | 1 | 0 | + | > 100 | 2.72E-03 |
| acetyl-CoA carboxylase activity (GO:0003989) | 4 | 1 | 0 | + | > 100 | 2.72E-03 |
| sequence-specific double-stranded DNA binding (GO:1990837) | 136 | 2 | 0.09 | + | 21.58 | 2.78E-03 |
| double-stranded DNA binding (GO:0003690) | 151 | 2 | 0.1 | + | 19.44 | 3.43E-03 |
| nucleic acid binding (GO:0003676) | 719 | 3 | 0.49 | + | 6.12 | 4.34E-03 |
| carboxyl- or carbamoyl transferase activity (GO:0016743) | 8 | 1 | 0.01 | + | > 100 | 5.44E-03 |
| sequence-specific DNA binding (GO:0043565) | 200 | 2 | 0.14 | + | 14.68 | 5.98E-03 |
| DNA-binding transcription factor activity (GO:0003700) | 228 | 2 | 0.16 | + | 12.87 | 7.74E-03 |
| transcription regulator activity (GO:0140110) | 249 | 2 | 0.17 | + | 11.79 | 9.20E-03 |
| mRNA binding (GO:0003729) | 22 | 1 | 0.01 | + | 66.71 | 1.49E-02 |
| Phosphor relay response regulator activity (GO:0000156) | 29 | 1 | 0.02 | + | 50.61 | 1.96E-02 |
| molecular transducer activity (GO:0060089) | 49 | 1 | 0.03 | + | 29.95 | 3.30E-02 |

**SI Table 3:** Interaction summary of nine shortlisted ligands with accD protein

| Ligand | H-bond | Other-non-bonded |
| --- | --- | --- |
| Tenapanor | HIS-A52, MET-A53, MET-A55, SER-A56, ARG-A60, THR-A102, GLU-A126, PHE-A127, SER-A128, THR-A199, ASP-A200, GLU-A220, PRO-A221, LYS-A222, ARG-A247, ARG-A263 | HIS-A51, ARG-A54, ARG-A58, ASN-A59, ALA-A161, PRO-A201, LYS-A240, PRO-A243, GLY-A244 |
| POPG | GLU-A234, PRO-A242, PRO-A243, GLY-A244, | SER-A29, CYS-A30, GLN-A32, LYS-A48, CYS-A49, HIS-A51, HIS-A52, MET-A53, ARG-A54, MET-A55, SER-A56, ALA-A57, ARG-A60, PHE-A125, GLU-A126, PHE-A127, SER-A128, THR-A199, ASP-A200, GLU-A220, PRO-A221, LYS-A222, ALA-A223, LEU-A224, PRO-A230, LEU-A241, PHE-A245, ARG-A247, ALA-A161, ARG-A263 |
| Deferoxamine | ARG-A41, SER-A63, TYR-A116, ARG-A263, PRO-A264, ARG-A267, VAL-A286, SER-A287, GLU-A288, PRO-A290, HIS-A291, VAL-A294, | TYR-A35, GLU-A38, LEU-A43, MET-A55, ARG-A60, LEU-A64, PHE-A159, GLU-A265, MET-A266, LEU-A268, LEU-A270, ALA-A285, ALA-A289, GLU-A292, GLY-A293, VAL-A296, |
| Lypressin | ASP-A28, ARG-A54, MET-A55, THR-A199, ASP-A206, PRO-A221, GLU-A234, ARG-A247, ARG-A263, | HIS-A51, HIS-A52, MET-A53, SER-A56, ARG-A60, PHE-A127, ALA-A161 PRO-A201, GLU-A220, LYS-A222, ALA-A223, LEU-A224, PRO-A230, ARG-A231 |
| Cobicistat | ARG-A41, SER-A63, LEU-A65, ASP-A66, GLU-A288 | GLU-38, ASN-A42, LEU-A43, PRO-A47, ARG-A60, HIS-A62, LEU-A64, GLU-A67, TYR-A116, PHE-A159, ARG-A263, PRO-A264, MET-A266, ARG-A267, LEU-A268, ALA-A285, VAL-A286, SER-A287, HIS-A291 |
| Viomycin | GLU-A40, HIS-A62, SER-A63, ASP-A66, GLU-A67, ARG-A263, GLU-A288 | ARG-A41, LEU-A43, MET-A55, ASN-A59, ARG-A60, LEU-A64, LEU-A65, GLY-A68, LEU-A70, PRO-A264, MET-A266, ARG-A267, LEU-A268, HIS-A291 |
| Micafungin | HIS-A51, HIS-A52, MET-A53, ASP-A200, GLU-A234, LYS-A240, PRO-A243, GLY-A244 | ASP-A28, ARG-A54, MET-A55, ARG-A60, THR-A199, GLU-A220, PRO-A221, LYS-A222, ALA-A223, LEU-A224, PRO-A230, LEU-A241, PRO-A242, PHE-A245, ARG-A247, ARG-A263 |
| Orlistat | SER-A29 | ASP-A28, HIS-A51, HIS-A52, MET-A53, ARG-A54, MET-A55, ARG-A60, ALA-A161, THR-A199, ASP-A200, GLU-A220, PRO-A221, LYS-A222, ALA-A223, LEU-A224, PRO-A243, GLY-A244, ARG-A247, GLU-A249, PHE-A250, ARG-A263 |
| Colfosceril palmitate | SER-A29, CYS-A49 | LYS-A48, ASP-A50, HIS-A51, HIS-A52, MET-A53, ARG-A54, MET-A55, SER-A56, ALA-A57, ARG-A60, ALA-A124, PHE-A125, GLU-A126, PHE-A127, PHE-A159, ALA-A161, THR-A199, ASP-A200, MET-A203, GLU-A220, PRO-A221, LYS-A222, ALA-A223, LEU-A224, GLU -A229, PRO-A230, ARG-A231, VAL-A232, GLN-A235, ARG-A247, ARG-A263, |

**SI Figure 1:** Subsystem category distribution of annotated genes in six *Klebsiella pneumoniae* strains based on RAST functional annotation. A) HS11286; B) ST11; C) ST307; D) ST16; E) VA585-22; F) ST231

**SI Figure 2**: Two-dimensional interaction diagrams of the docked ligands with the target. (A): 1-Palmitoyl-2-oleoyl-sn-glycero-3-(phospho-rac-(1-glycerol)) (B): Cobicistat (C): Colfosceril palmitate (D): Deferoxamine (E): Lypressin (F): Orlistat (G): Tenapanor (H): Viomycin (I): Micafungin

**Comparative Hydrogen-Bond Occupancy Analysis of accD-Ligand Complexes**

Hydrogen-bond occupancy analysis was performed on the accD-ligand complexes throughout the 100 ns molecular dynamics simulations to evaluate the persistence and stability of intermolecular interactions. Hydrogen bonds exhibiting high occupancy percentages were considered indicative of stable contacts that contribute significantly to ligand retention within the binding pocket. The analysis revealed distinct interaction patterns among the seven investigated complexes, ranging from highly persistent hydrogen-bond networks to transient interactions.

**Cobicistat**

The accD-Cobicistat complex exhibited a strong hydrogen-bonding profile dominated by interactions with Arg262 and Arg41. The most persistent hydrogen bond was observed between ARG262 (NH2) and Cobicistat N32, showing an occupancy of approximately 70.2%, followed by ARG262 (NH1)-N49 (28.6%) and ARG41 (NH2)-O18 (20.8%). Additional contacts involving ASN42, HIS291, GLU265, and SER287 displayed lower occupancies (<15%), suggesting intermittent stabilization throughout the simulation. The predominance of ARG262-mediated interactions indicates that this residue plays a major role in anchoring Cobicistat within the binding cavity.

**SI Figure 3:** Hydrogen-bond occupancy analysis of the accD-Cobicistat complex during the 100 ns molecular dynamics simulation.

**Deferoxamine**

The accD-Deferoxamine complex formed an extensive hydrogen-bond network involving residues ARG41, GLU265, HIS291, ARG267, LEU268, ASN42, and GLU288. Multiple donor–acceptor pairs contributed to ligand stabilization, indicating a highly interactive binding mode. Particularly notable were recurring contacts involving ARG41, GLU265, and HIS291, which persisted throughout substantial portions of the trajectory. The presence of numerous interaction sites suggests that Deferoxamine remains well accommodated within the binding pocket through cooperative hydrogen-bonding interactions.

**SI Figure 4:** Hydrogen-bond occupancy analysis of the accD-Deferoxamine complex during the 100 ns molecular dynamics simulation.

**Micafungin**

Micafungin exhibited the strongest and most persistent hydrogen-bond network among all investigated compounds. The dominant interactions were GLY244 (N)-Micafungin N70 (92.81%) and GLN246 (NE2)-Micafungin N1 (79.90%), indicating exceptionally stable contacts maintained for the majority of the simulation period. Additional interactions with PHE245, LYS240, ARG231, ARG247, and ARG54 further reinforced ligand binding. The very high occupancies observed for the principal hydrogen bonds suggest a highly stable binding mode and strong complementarity between Micafungin and the accD active site.

**SI Figure 5:** Hydrogen-bond occupancy analysis of the accD-Micafungin complex during the 100 ns molecular dynamics simulation.

**POPG**

The POPG complex displayed a comparatively weak hydrogen-bonding pattern, with all interactions showing occupancies below approximately 15%. The most persistent contacts involved ARG247, TRP3, PHE245, LYS240, and LYS222. Although several hydrogen bonds were observed throughout the simulation, their relatively low occupancies indicate transient formation and dissociation events. This behavior suggests that POPG stabilization within the binding pocket is likely supported by other non-covalent interactions in addition to hydrogen bonding.

**SI Figure 6:** Hydrogen-bond occupancy analysis of the accD-POPG complex during the 100 ns molecular dynamics simulation.

**Tenapanor**

The accD-Tenapanor complex demonstrated a well-organized hydrogen-bond network characterized by several moderate-to-high occupancy interactions. The most persistent contact was formed between ARG247 (NH2) and Tenapanor O34, exhibiting an occupancy of approximately 62.17%. Additional stabilizing interactions included HIS51 (NE2)-N32 (40.17%) and ARG231 (NE)-O26 (26.83%), together with multiple contacts involving ARG54, ARG58, and ARG60. The presence of several persistent arginine-mediated hydrogen bonds indicates robust stabilization of Tenapanor within the binding pocket.

**SI Figure 7:** Hydrogen-bond occupancy analysis of the accD–Tenapanor complex during the 100 ns molecular dynamics simulation.

**Viomycin**

Viomycin formed a stable hydrogen-bonding network involving residues distributed across the binding cavity. The strongest interactions were ALA123 (N)-O43 (51.35%), GLY164 (N)-O34 (46.89%), THR199 (N)-N14 (42.01%), and ARG60 (NE)-N45 (38.97%). Several additional interactions with PHE159 and GLY132 contributed to ligand stabilization. The relatively high occupancies of multiple hydrogen bonds suggest that Viomycin maintains a favorable and stable orientation within the active site during the simulation.

**SI Figure 8:** Hydrogen-bond occupancy analysis of the accD-Viomycin complex during the 100 ns molecular dynamics simulation.

**Lypressin**

The accD-Lypressin complex exhibited the weakest hydrogen-bonding profile among the analyzed ligands. The most persistent interaction was Lypressin N24-ASP50 (OD1) with an occupancy of 9.81%, while additional contacts involving ARG231 and SER29 displayed occupancies of only 3-4%. The low occupancy values indicate highly transient hydrogen-bond formation and suggest that ligand stabilization is not primarily driven by persistent hydrogen-bond interactions.

**SI Figure 9:** Hydrogen-bond occupancy analysis of the accD-Lypressin complex during the 100 ns molecular dynamics simulation.

(a)

(b)

(d)

(c)

(e)

(f)

(h)

(g)

(g)

(h)

**SI Figure 10:** Principal component analysis (PCA)-based Free Energy Landscapes (FELs) of the native accD protein and ligand-bound complexes generated from 100 ns molecular dynamics simulations. (a) Cobicistat, (b) Deferoxamine, (c) Lypressin, (d) Micafungin, (e) Native accD, (f) 1-Palmitoyl-2-oleoyl-sn-glycero-3-(phospho-rac-(1-glycerol)) (POPG), (g) Tenapanor, and (h) Viomycin

Two-dimensional free energy landscape representations were constructed using the first two principal components (PC1 and PC2) as reaction coordinates. The 2D contour maps illustrate the conformational space sampled during the simulation, Dark blue regions represent low-energy minima corresponding to thermodynamically favourable and stable conformations, whereas lighter green to yellow regions indicate higher-energy and less populated conformational states. The compactness and distribution of the low-energy basins reflect differences in the conformational stability and dynamic behaviour of the respective protein–ligand complexes.
